# Hydrogen Gas Enhances Salinity Tolerance in Tomato Seedlings by Regulating the *S*‐Nitrosylation of MEK1

**DOI:** 10.1111/pbi.70585

**Published:** 2026-02-18

**Authors:** Hua Fang, Xuetong Wu, Dengjing Huang, Xinfang Chen, Zhiya Liu, Xuejuan Pan, Huan Chen, Dandan Zheng, Caicai Ma, Xuemei Hou, Shuya Wang, Chunlei Wang, Weibiao Liao

**Affiliations:** ^1^ College of Horticulture, Gansu Agricultural University Lanzhou China; ^2^ State Key Laboratory of Aridland Crop Science Gansu Agricultural University Lanzhou China

**Keywords:** hydrogen gas, MEK1 protein, protein interactions, salt stress, site mutation, *S*‐nitrosylation

## Abstract

Hydrogen gas (H_2_) effectively alleviates abiotic stress in horticultural plants. Protein *S*‐nitrosylation, a key post‐translational modification, serves as a critical mechanism for nitric oxide (NO) to exert its biological function under adverse conditions. However, the relationship among H_2_, NO and *S*‐nitrosylation in response to salt stress remains poorly understood. In this study, we demonstrate that NO participated in H_2_‐enhanced salt tolerance in tomato seedlings ( *Solanum lycopersicum* cv. Micro‐Tom). H_2_ triggered an increase in NO levels and *S*‐nitrosothiol (SNO) content under salt stress, enhancing the enrichment of *S*‐nitrosylated proteins. *S*‐nitrosoproteomic analysis revealed that MEK1, a conserved MAPK component, was notably induced and *S*‐nitrosylated by NO and H_2_ under salt stress. Furthermore, *SlMEK1*‐overexpressing tomato lines exhibited enhanced salt stress tolerance, while knockout lines showed reduced tolerance, indicating the positive regulatory role of *SlMEK1* in salt tolerance. Additionally, *SlMEK1* may contribute to H_2_‐enhanced salt tolerance. Meanwhile, MEK1 was shown to undergo *S*‐nitrosylation at Cys 172, and this modification was involved in H_2_‐enhanced salinity tolerance. Moreover, site‐specific mutation analysis at Cys 172 confirmed that MEK1 *S*‐nitrosylation positively contributed to both intrinsic and H_2_‐facilitated salt tolerance. Finally, *S*‐nitrosylation of MEK1 increased its interaction with GSNOR, and H_2_ further promoted this interaction under salt stress. Collectively, our findings indicate that H_2_ may enhance the salinity tolerance in tomato seedlings by orchestrating the interplay between *S*‐nitrosylated MEK1 and GSNOR.

## 1 Introduction

Salt stress, a major environmental constraint, restricts plant ecosystem distribution, hampers crop development and threatens global food security (van Zelm et al. 2020). It induces toxic ion accumulation in plant cells, impairing metabolic functions and suppressing growth (Yang and Guo 2018). To counteract these detrimental effects, plants employ multiple defence mechanisms, including the activation of Mitogen‐activated protein kinases (MAPKs/MPKs), the upregulation of antioxidant enzyme systems and the efflux of toxic ions (Yang and Guo 2018; Yu et al. 2010; Rus et al. 2004). MAPKs, as key signalling modules downstream of cell surface receptors, relay signals from ligand‐receptor interactions to intracellular events through substrate phosphorylation (Lucrezi et al. 2014). The diversity of MAPK substrates, coupled with their spatiotemporal expression patterns, enables MAPK cascades to regulate a wide range of biological processes (Xu and Zhang 2015; Komis et al. 2018; Zhang, Su, et al. 2018).

Hydrogen gas (H_2_), a newly identified gaseous signalling molecule, plays a beneficial role in alleviating abiotic stress across a wide range of plant species. Hydrogen‐rich water (HRW, an H_2_ donor) has been shown to alleviate salt stress‐induced reactive oxygen species (ROS) by downregulating cucumber protein kinase ROP1, thereby reducing oxidative damage (Yu et al. 2021). H_2_ also helps maintain intracellular ionic homeostasis. In barley, HRW increased Na^+^ efflux through the root epidermal SOS1 protein kinase Na^+^/H^+^ exchanger while reducing the sensitivity of K^+^ efflux channels to ROS. This resulted in an improved Na^+^/K^+^ ratio, re‐established ionic homeostasis and enhanced salt tolerance (Davletova et al. 2005; Wu et al. 2020). Additionally, Xie et al. (2015) showed that HRW enhanced alfalfa's tolerance to ultraviolet b (UV‐B)‐induced oxidative damage. Moreover, HRW reduced nitrate reductase‐mediated nitric oxide (NO) production and mitigated aluminium (Al) toxicity's inhibitory effect on alfalfa root elongation (Chen et al. 2014). Investigating the broader mechanisms by which H_2_ mitigates abiotic stress could provide valuable insights for improving plant tolerance and ensuring normal growth.

As second messengers, NO and H_2_ interact extensively during plant development and abiotic stress responses. Zhu et al. (2016) proposed that NO may act as a downstream signalling molecule in H_2_‐induced adventitious root development in cucumber. Similarly, this interaction was observed in mitigating cadmium (Cd) toxicity in *Brassica campestris* seedlings, where HRW induced H_2_ accumulation and stimulated NO synthesis under Cd stress, synergistically enhancing seedling tolerance (Su, Wu, et al. 2019). Interestingly, NO mediated H_2_‐induced adventitious root formation by regulating plasma membrane H^+^‐ATPase and 14–3‐3 protein expression and their interactions, revealing another regulatory mechanism for NO and H_2_ interplay (Li et al. 2020). Notably, Su et al. (2018) reported that NO participated in H_2_‐triggered osmotic tolerance by promoting proline synthesis and re‐establishing redox homeostasis. Importantly, NO‐dependent *S*‐nitrosylation may mediate H_2_‐triggered osmotic tolerance, underscoring a potential regulatory mechanism (Su et al. 2018). Evidently, although multiple relationships between H_2_ and NO have been identified, direct evidence for the regulatory mechanisms of their interactions during plant salt stress remains limited.

The regulatory mechanism of NO is primarily mediated by post‐translational modification (PTMs), particularly protein *S*‐nitrosylation. In this process, an NO molecule attaches to reactive cysteine (Cys or C) residues of target proteins, forming S‐nitrosothiols (SNO) (Liu et al. 2023). In plants, NO modulates protein activity, subcellular localisation and conformational changes through *S*‐nitrosylation, effectively regulating plant growth, development, stress response and hormonal signalling (Zhang et al. 2019; Zhang and Liao 2019; Lin et al. 2023). Despite NO's emerging role as a key regulator of plant signalling and abiotic stress responses via *S*‐nitrosylation, only 23 *S*‐nitrosylated proteins have been functionally characterised in plant species to date (Feng et al. 2019). Research has demonstrated that *S*‐nitrosylation directly contributes to stress tolerance by fine‐tuning the activities of key proteins. NO enhanced GTPase activity via *S*‐nitrosylation of the GTPase RABG3E (RAB7) at Cys 171, a modification that promotes vesicular transport. This process also strengthened the interaction of RAB7 with phosphatidylinositol‐4‐phosphate (PI4P), ultimately enhancing salt tolerance in *Arabidopsis thaliana* (Lin et al. 2023). Additionally, *S*‐nitrosylation of 1‐aminocyclopropane‐1‐carboxylic acid oxidase homologue 4 (ACOh4) at Cys172 activates its enzymatic function, enabling ACOh4 to regulate Na^+^ and H^+^ efflux, maintain K^+^/Na^+^ homeostasis and promote the transcription of salt‐tolerant genes, thereby enhancing salt tolerance in tomato (Liu et al. 2023). *S*‐nitrosylation of Δ1‐pyrroline‐5‐carboxylic acid reductase (SlP5CR) at Cys 5 improved its NAD(P)H affinity and enzyme activity, enhancing drought and salt tolerance in tomato (Liu, Wei, et al. 2024). Under saline‐alkali stress, melatonin alleviated *S*‐nitrosylation‐mediated inhibition of plasma membrane H^+^‐ATPase (HA2) by scavenging excess NO, restoring its proton‐pumping function and cellular ion homeostasis (Wei et al. 2025). Additionally, NO differentially regulates the *S*‐nitrosylation of SlGABA‐TP enzymes in a tissue‐specific manner, balancing stress resilience in roots and quality development in fruits (Liu, Cao, et al. 2024). Collectively, these findings highlight that *S*‐nitrosylation serves as a molecular foundation for comprehensive plant stress resistance by regulating diverse physiological processes, including vesicular trafficking, ion homeostasis and metabolic pathways. While initial insights into *S*‐nitrosylation of target proteins have been achieved, further identification of *S*‐nitrosylated proteins and investigation of their mechanisms remain essential.

As outlined above, the regulatory roles of H_2_ and NO in plant growth and stress responses have been well‐characterised. Protein *S*‐nitrosylation is recognised as a key mechanism through which NO mediates its biological functions during stress responses. While some evidence exists regarding the interaction between NO and MAPK signalling in plants, the *S*‐nitrosylation of MAPKs under abiotic stress conditions remains largely unexplored. Furthermore, the complex interplay among H_2_, NO, protein S‐nitrosylation and MAPKs in salt stress adaptation has yet to be elucidated. This study unveils a novel mechanism by which H_2_ enhances salt tolerance in tomato ( *Solanum lycopersicum* L. cv ‘Micro Tom’) seedlings by modulating the *S*‐nitrosylation of MEK1, marking a significant advancement in our understanding of gas signalling and post‐translational regulation under abiotic stress. Through a multidisciplinary approach—including physiological, molecular and proteomic analyses—we elucidate how H_2_ mitigates salt stress via MEK1 *S*‐nitrosylation. This discovery not only highlights the critical role of protein *S*‐nitrosylation in stress adaptation but also significantly advances our knowledge of gas signalling and post‐translational regulation in plants.

## 2 Results

### 2.1 NO Participates in H_2_ ‐Enhanced Salinity Tolerance in Tomato Seedlings

H_2_ has been shown to enhance plant resistance to abiotic stresses (Jin et al. 2013; Xie et al. 2014). To investigate whether H_2_ can mitigate salt stress in tomato seedlings, we treated the plants with hydrogen‐rich water (HRW), a donor of H_2_, under salt stress conditions. Compared to the control, NaCl treatment significantly inhibited the growth of tomato seedlings (Figure S1a–c). HRW alleviated the inhibitory effect of salt stress in a dose‐dependent manner, as evidenced by improvements in plant height, shoot dry weight, shoot fresh weight and total root length (Figure S1b,d,f,i). Notably, among the tested concentrations, 75% HRW was the most effective, restoring plant height, stem diameter, shoot and root fresh weight, shoot and root dry weight, leaf area and total root length to control levels (Figure S1b–k). Due to its pronounced efficacy, 75% HRW was selected for further investigation.

**FIGURE 1:**
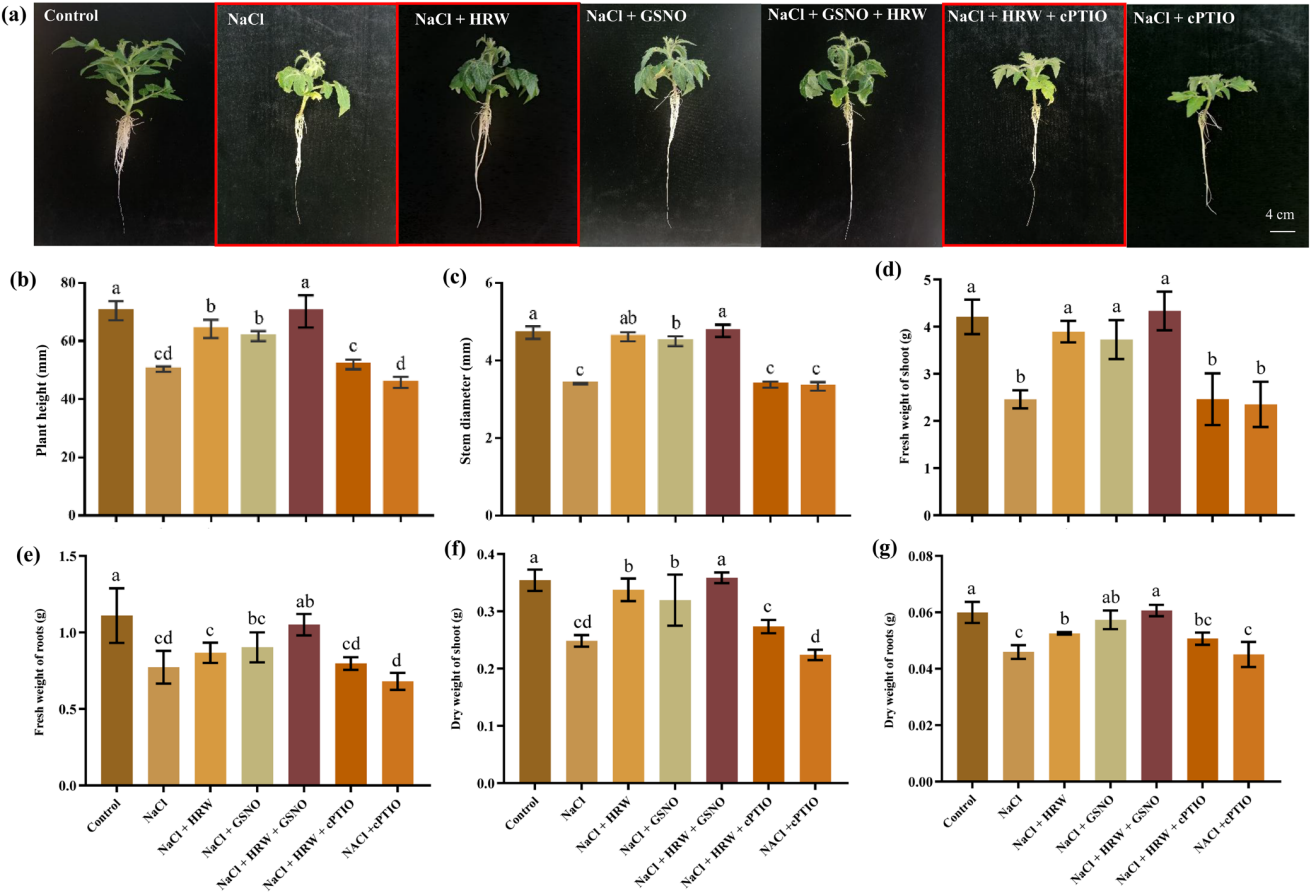
Effect of NO scavenger cPTIO on HRW‐induced salt tolerance in tomato seedlings. Phenotypic analysis of tomato seedlings (a), plant height (b), stem diameter (c), fresh weight of shoot (d), fresh weight of roots (e), dry weight of shoot (f) and dry weight of roots (g) under control, NaCl, NaCl + HRW, NaCl + GSNO, NaCl + HRW + GSNO and NaCl + HRW + cPTIO treatments. Measurements were taken 7 days post‐treatment. Data are expressed as mean ± SD (*n* = 3), and experiments were performed in triplicate. Different letters indicate significant differences (*p* < 0.05), as determined by Duncan's multiple range test.

NO is a key regulator of multiple developmental processes and stress responses in plants, acting as a downstream molecule in the H_2_ signalling pathway (Zhu et al. 2016). To determine whether NO is involved in H_2_‐enhanced salinity tolerance, HRW, GSNO (a NO donor) and 2‐(4‐carboxyphenyl)‐4,4,5,5‐tetramethylimidazoline‐1‐oxyl‐3‐oxide (cPTIO, a NO scavenger) were utilised in the same experimental system (Figure 1 and Figure S2). Compared to NaCl alone, NaCl + HRW and NaCl + GSNO increased plant height, stem diameter, shoot and root fresh weight, shoot and root dry weight, leaf area and total root length in tomato seedlings (Figure 1b–g; Figure S2). Co‐treatment with HRW and GSNO further enhanced these traits under salt stress (Figure 1b–g), indicating a synergistical interaction between NO and H_2_ in promoting salt tolerance. Conversely, NaCl + HRW + cPTIO significantly reduced plant height, stem diameter, shoot and root fresh weight and shoot and root dry weight compared to NaCl + HRW (Figure 1b–g). Similar trends were observed for root length and leaf area (Figure S2). The reversal of HRW's beneficial effects by the NO scavenger cPTIO demonstrates that NO plays a critical role in H_2_‐enhanced salt tolerance.

**FIGURE 2:**
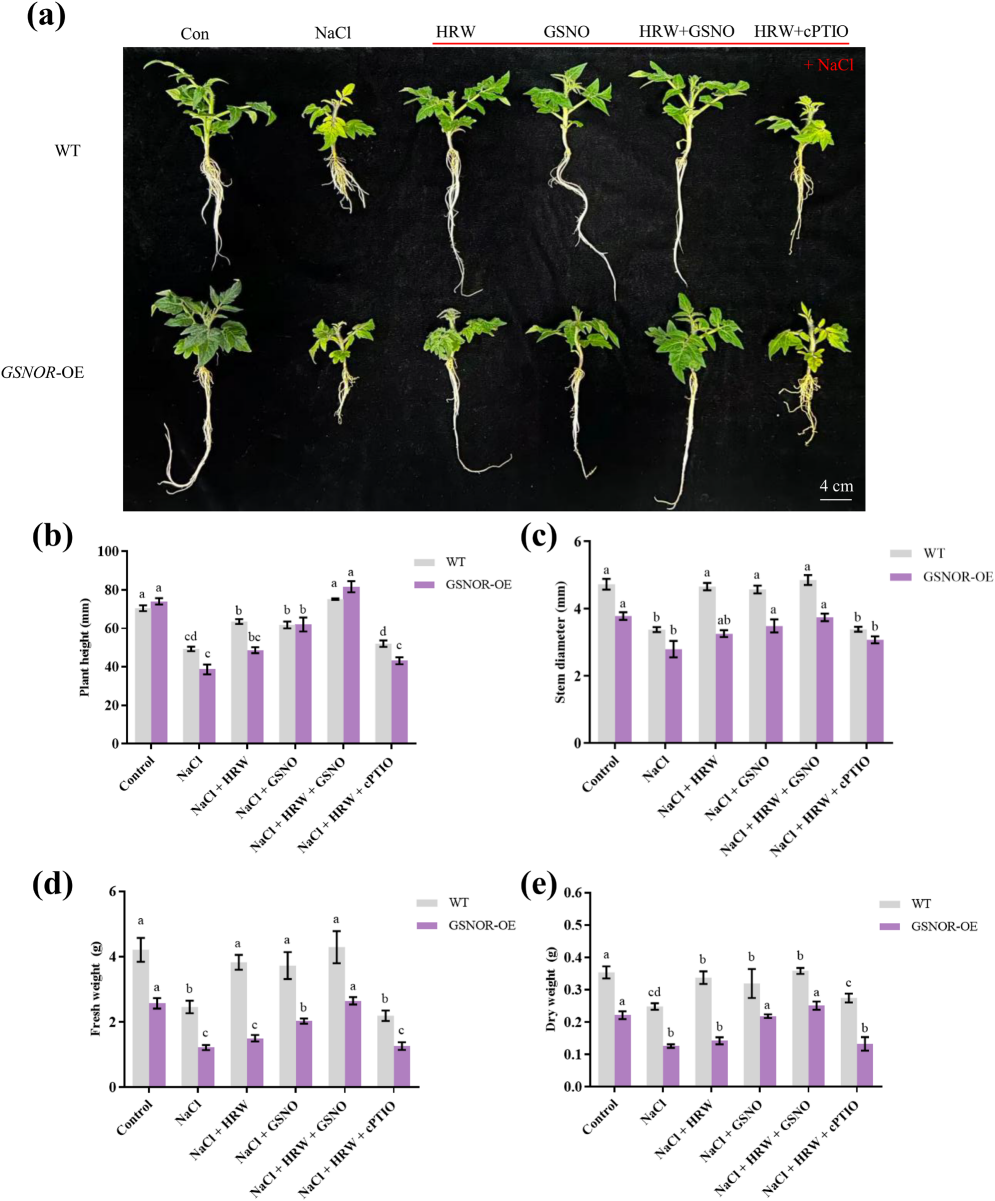
Effect of *SlGSNOR* overexpression (*SlGSNOR*‐OE) on HRW‐induced salt tolerance in tomato seedlings. Phenotypic analysis of WT and *SlGSNOR‐OE* (a), plant height (b), stem diameter (c), fresh weight (d) and dry weight (e) under control, NaCl, NaCl + HRW, NaCl + GSNO, NaCl + HRW + GSNO and NaCl + HRW + cPTIO treatments. Measurements were taken 7 days post‐treatment. Data are expressed as mean ± SD (*n* = 3), and experiments were performed in triplicate. Different letters indicate significant differences (*p* < 0.05), as determined by Duncan's multiple range test.

Gong et al. (2019) have shown that knockdown of *S*‐*nitrosoglutathione reductase* (*GSNOR*) elevated endogenous NO levels. To further elucidate the relationship between H_2_ and NO in alleviating salt stress in tomato seedlings, 4‐week‐old *SlGSNOR* overexpression transgenic seedlings were treated with NaCl, NaCl + HRW, NaCl + GSNO, NaCl + HRW + GSNO and NaCl + HRW + cPTIO. Compared to WT, *SlGSNOR*‐OE lines exhibited severe growth inhibition under NaCl stress (Figure 2a–e), suggesting that lower NO levels in *SlGSNOR*‐OE seedlings may impair salt resistance. Additionally, HRW treatment failed to alleviate the inhibition of plant height, stem diameter, fresh weight, dry weight, leaf area and total root length in *SlGSNOR*‐OE lines under salt stress (Figure 2b–e, Figure S3a–d), consistent with the inhibitory effects of the NO scavenger cPTIO on HRW‐induced salt tolerance (Figure 1). However, exogenous GSNO significantly mitigated the reduction in plant height, stem diameter, fresh weight and dry weight under salt stress (Figure 2b–e). These results suggest that NO is essential for H_2_‐enhanced salinity tolerance, as reduced endogenous NO levels reversed the beneficial effects of HRW. Therefore, these findings provide robust evidence that NO and H_2_ are critical molecules that interact synergistically to improve salinity tolerance.

### 2.2 H_2_ ‐Induced NO Triggers Protein *S*‐Nitrosylation in Tomato Seedlings Under Salt Stress

**FIGURE 3:**
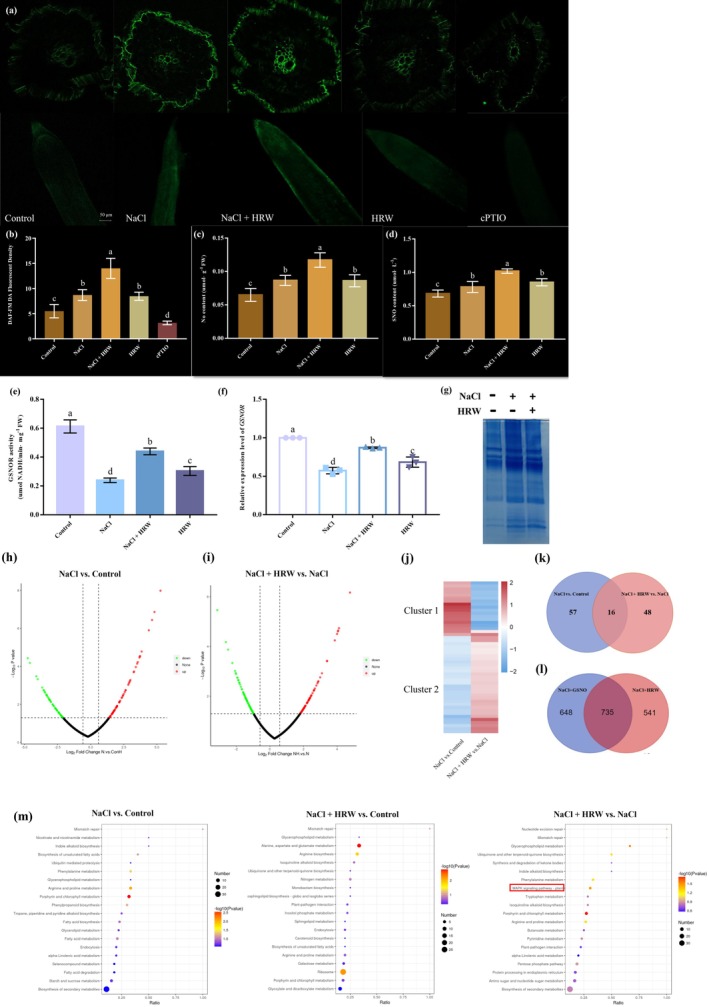
Identification and analysis of *S*‐nitrosylated proteins in tomato seedling. H_2_‐induced endogenous NO production (a‐c) and total *S*‐nitrosothiol (SNO) production (d) under NaCl, NaCl+HRW and HRW treatments. *S*‐nitrosoglutathione reductase (GSNOR) activity (e) and *GSNOR* expression levels (f) during HRW‐enhanced salinity tolerance. MS‐based detection of *S*‐nitrosylated proteins (colloidal CBB‐stained 12% SDS PAGE gel) (g). LC–MS/MS‐based Volcano plots of *S*‐nitrosylated proteins in ‘NaCl vs. Control’ and ‘NaCl + HRW vs. NaCl’ (h, i). The dotted lines on the x‐ and y‐axes represent the fold‐change = 1.2 and *p* = 0.05, respectively. Heat map analysis of *S*‐nitrosylated proteins in ‘NaCl vs. Control’ and ‘NaCl + HRW vs. NaCl’ (j). Venn diagram of all upregulated *S*‐nitrosylated proteins in ‘NaCl vs. Control’ and all downregulated *S*‐nitrosylated proteins in ‘NaCl + HRW vs. NaCl’ (k). Venn diagram of *S*‐nitrosylated proteins in ‘NaCl + HRW vs NaCl + GSNO’ (l). KEGG enrichment analysis of *S*‐nitrosylated proteins (m).

Previous study has shown that exposure to HRW can elevate NO levels (Zhu et al. 2016). A primary function of NO in biological systems is its involvement in protein *S*‐nitrosylation. To explore the potential role of NO in H_2_‐enhanced salinity tolerance, we investigated the effect of HRW on endogenous NO content and protein *S*‐nitrosylation under salt stress (Figure 3a–g). Quantification analysis revealed that NaCl, HRW and NaCl + HRW treatments significantly increased the accumulation of SNOs and NO (Figure 3a–d). To further elucidate the involvement of protein *S*‐nitrosylation in H_2_‐ mediated alleviation of salt stress, GSNOR activity and *GSNOR* expression levels were measured (Figure 3e,f). The increased accumulation of SNOs and NO under NaCl, HRW and NaCl + HRW treatments resulted in GSNOR deficiency (Figure 3e,f), potentially disrupting *S*‐nitrosylation equilibrium. Additionally, we employed a biotin‐switch assay to quantify the levels of *S*‐nitrosylated proteins. Significantly higher levels of *S*‐nitrosylated proteins were detected in NaCl and NaCl + HRW treatments compared to the control (Figure 3g). These findings indicate that both salinity stress and H_2_‐enhanced salinity tolerance processes are associated with an elevated degree of protein *S*‐nitrosylation.

To further explore the mechanisms underlying protein *S*‐nitrosylation during H_2_‐alleviated salt stress, we utilised the biotin‐switch method coupled with LC–MS/MS to identify *S*‐nitrosylated proteins using a proteomic approach. The reliability of the experimental data was confirmed by the mass error distribution and the length distribution of the identified peptides (Figure S4a,b). The molecular masses of the identified proteins were predominantly within the 20–60 kDa range, aligning with the enriched zone of *S*‐nitrosylated proteins observed in the gel (Figure S4c; Figure 3g). Furthermore, the lengths of the enriched peptides were primarily distributed between 7 and 25 amino acids (Figure S4d), further validating the robustness of the mass spectrometry data. Subcellular localization analysis indicated that the *S*‐nitrosylated proteins were mainly localised in chloroplasts, cytoplasm, mitochondrion and nucleus (Figure S4e–g). Mass spectrometry analysis identified 1186 proteins that were constitutively *S*‐nitrosylated across all three treatments (Figure S4h). Among these, 58 of *S*‐nitrosylated proteins were uniquely identified in the control, while 5 were specific to NaCl + HRW treatment, highlighting their potential significance in *S*‐nitrosylation mechanisms underlying H_2_‐mediated salt stress responses. Notably, no unique *S*‐nitrosylated proteins were detected in the NaCl treatment alone (Figure S4h).

To study the roles of protein *S*‐nitrosylation in biological processes, we performed a Gene Ontology (GO) analysis. The results showed similar distributions across the control (Con), NaCl and NaCl + HRW treatments, with proteins primarily involved in oxidation–reduction, translation and metabolic processes (Figure S5a–c), suggesting that *S*‐nitrosylation regulates basal metabolism. Functional classification further indicated that most differential proteins were associated with ‘translation, ribosomal structure and biogenesis,’ ‘posttranslational modification’ and ‘protein turnover, chaperones’ (Figure S5d–f), indicating their potential role in H_2_‐mitigated alleviation of salt stress.

In the comparison of ‘NaCl vs. Control’, 73 *S*‐nitrosylated proteins were significantly upregulated, while 66 significantly downregulated, indicating that saline stress enhances proteome *S*‐nitrosylation (Figure 3h). Similarly, in the comparison of ‘NaCl + HRW vs. NaCl’, 72 *S*‐nitrosylated proteins were significantly upregulated, while 64 were downregulated, suggesting the regulatory role of H_2_ in *S*‐nitrosylation under stress conditions (Figure 3i). To visualise the regulatory patterns of protein expression across treatments, we conducted hierarchical cluster analysis on the significantly differentially expressed proteins in both comparisons (NaCl vs. Control and NaCl + HRW vs. NaCl). The resulting heat map revealed 2 distinct clusters (Figure 3j). Cluster 1 represents a group of proteins induced by salt stress but suppressed or normalised by HRW treatment (Figure 3j). These findings collectively demonstrate that H_2_ alleviates saline stress‐triggered proteome *S*‐nitrosylation.

Venn analysis of differentially *S*‐nitrosylated proteins identified 16 core targets that were upregulated under salt stress but downregulated following HRW treatment, suggesting their pivotal role in H_2_‐mediated salt stress mitigation (Figure 3k). GO enrichment analysis revealed that salt stress primarily enriched biological processes related to stress response (e.g., ‘response to wounding’, ‘response to stimulus’) and metabolic processes (e.g., ‘fatty acid metabolic process’, ‘protein folding’), indicating comprehensive metabolic reprogramming and activation of damage repair mechanisms in response to osmotic and ionic imbalance (Figure S6a). In contrast, HRW shifted the cellular response toward homeostasis recovery and energy regulation, with specific enrichment of functions such as ‘ATP binding’, implying that maintaining cellular energy status is a key mechanism underlying HRW‐induced stress alleviation (Figure S6b). Additionally, both salt stress and HRW treatment co‐enriched fundamental protective functions, including ‘photosystem II stabilization’ (Figure S6a,b). These findings suggest that HRW contributes to a systemic alleviation mechanism by reversing the salt‐induced suppression of these protective functions while synergizing with its unique role in energy regulation.

To explore the key pathways of *S*‐nitrosylated proteins in H_2_‐alleviated salt stress alleviation, we performed KEGG pathway analysis for Con vs. NaCl, Con vs. NaCl + HRW and NaCl vs. NaCl + HRW (Figure 3m). In the Con vs. NaCl comparison, *S*‐nitrosylated proteins were enriched in porphyrin and chlorophyll metabolism and phenylalanine metabolism. In Con vs. NaCl + HRW, they were primarily involved in alanine, aspartate and glutamate metabolism, ribosome function and arginine biosynthesis. In the NaCl vs. NaCl + HRW comparison, they were associated with porphyrin and chlorophyll metabolism, glycerophospholipid metabolism, mitogen‐activated protein kinase (MAPK) signalling, phenylalanine metabolism, arginine and proline metabolism, nucleotide excision repair and mismatch repair. Notably, glycerophospholipid metabolism, MAPK signalling, arginine and proline metabolism, nucleotide excision repair and mismatch repair were uniquely identified in the NaCl vs. NaCl + HRW comparison.

The MAPK cascades are key signalling pathways in eukaryotic cells, translating extracellular signals into intracellular responses via phosphorylation. However, the role of *S*‐nitrosylation in MAPK pathway proteins remains unexplored. Here, we analysed *S*‐nitrosylated proteins within the MAPK pathway (Figure S6c and Table 1) and identified eight such proteins (Table 1), suggesting their involvement in H_2_‐alleviated salt stress. These findings open a new avenue for future research on *S*‐nitrosylation in MAPK pathway‐related proteins during stress responses.

**TABLE 1:** The identified differential *S*‐nitrosylated proteins involved in MAPK pathway in the NaCl vs. NaCl + HRW comparison.

Accession number	Protein name	Gene name
K4BAE6	Catalase	*CAT1*
A0A3Q7GYF2	Nucleoside diphosphate kinase	*NDPK2*
Q05538	Basic 30 kDa endochitinase	*CHI9*
A0A3Q7IHS3	Chitin‐binding type‐1 domain‐containing protein	*CHIB*
O48616	MAP kinase kinase	*MEK1*
A0A3Q7HXW1	Uncharacterized protein	*PR1*
A0A3Q7EQ44	Protein kinase domain‐containing protein	*SNRK2*
K4DI20	Uncharacterized protein	*CALM*

To study the role of H_2_‐triggered NO in inducing protein *S*‐nitrosylation, we performed a VENN analysis of proteins under NaCl + HRW and NaCl + GSNO treatments. Data for NaCl + GSNO were sourced from our previous study (Wei et al. 2022). We identified 1924 putative *S*‐nitrosylated proteins shared between the two treatments (Figure 3L), including 735 coexisting proteins (Table S1), 15 of which are implicated in the MAPK signalling pathway (Table 2).

**TABLE 2:** MAPK pathway‐associated *S*‐nitrosylation modification proteins commonly shared in the NaCl+HRW and NaCl+GSNO comparison.

Accession number	Protein name	Gene name
K4BAE6	Catalase	*CAT1*
A0A3Q7GYF2	Nucleoside diphosphate kinase	*NDPK2*
Q05538	Basic 30 kDa endochitinase	*CHI9*
A0A3Q7IHS3	Chitin‐binding type‐1 domain‐containing protein	*CHIB*
O48616	MAP kinase kinase	*MEK1*
P93212	14–3‐3 protein 7	*TFT7*
P93214	14–3‐3 protein 9	*TFT9*
A0A3Q7EQ44	Protein kinase domain‐containing protein	*SNRK2*
A0A3Q7IMD9	S‐adenosylmethionine synthase	*101 245 012*
P43282	S‐adenosylmethionine synthase 3	*SAM3*
P43280	S‐adenosylmethionine synthase 1	*SAM1*

### 2.3 *SlMEK1* Is Involved in H_2_ ‐Enhanced Salt Tolerance in Tomato Seedings

Our previous study found that the expression levels of *SlMEK1* were significantly up‐regulated by GSNO treatment under salt stress (Wei et al. 2022). This observation led us to explore whether MEK1 also contributes to H_2_‐enhanced salt tolerance. The results revealed that *SlMEK1* responded robustly to salt stress (Figure S7a), with elevated expression under NaCl + HRW treatment. Consistently, the protein level of MEK1 increased in response to HRW under salt stress (Figure S7b). Compared to NaCl treatment alone, a continued but less pronounced increase was observed under NaCl + HRW treatment. As shown in Table 2, MEK1 may also be a potential *S*‐nitrosylation candidate during H_2_‐enhanced salinity tolerance.

To determine the roles of *SlMEK1* in H_2_‐enhanced salt tolerance, 3 independent *slmek1* mutants (*mek1‐2*, *mek1‐7* and *mek1‐12*) were generated using the CRISPR/cas9 system (Figure S8). *SlMEK1* knockout caused a base deletion in the gene sequence in the *mek1* lines. Specifically, the *mek1‐2*, *mek1‐7* and *mek1‐12* lines exhibited −1 bp, −4 bp and −2 bp deletions in their exon regions, respectively. These T2 plants displayed similar phenotypes, and 3 knockout lines were validated using qRT‐PCR (Figure S8a,b). Consequently, three representative lines were treated, and the phenotype of the *mek1‐2* mutant is shown.

**FIGURE 4:**
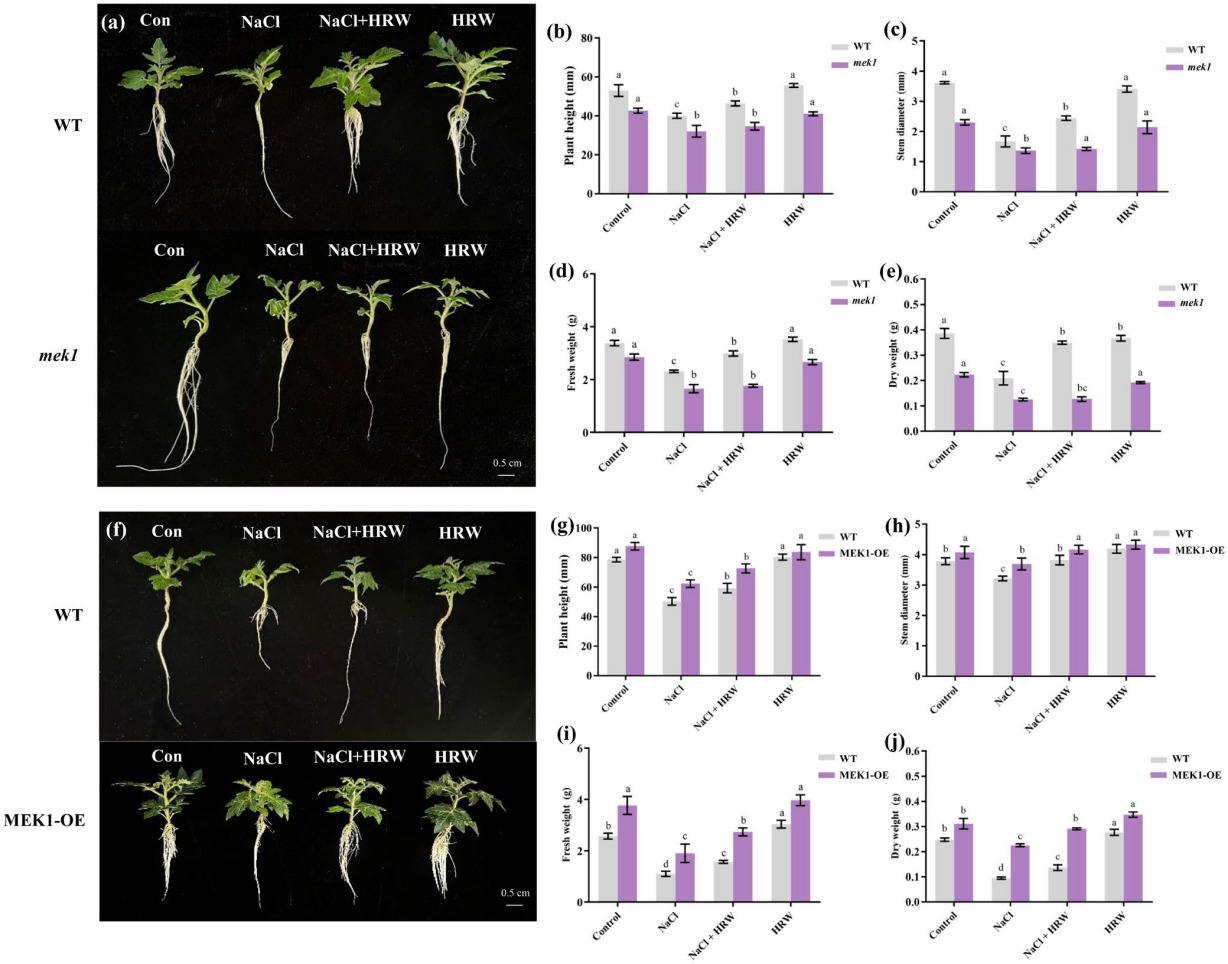
Assessment of *SlMEK1* genetic materials on HRW‐induced salt tolerance in tomato seedlings. Phenotypic analysis of *SlMEK1* knockout lines (a). Evaluation of *mek1* mutant under control, NaCl, NaCl + HRW and HRW treatments, including plant height (b), stem diameter (c), fresh weight (d) and dry weight (e). Phenotypic analysis of *SlMEK1* overexpressing lines (f). Evaluation of OE‐*SlMEK1* lines under control, NaCl, NaCl + HRW and HRW treatments, including plant height (g), stem diameter (h), fresh weight (i) and dry weight (j). Measurements were taken 7 d post‐treatment. Data are expressed as mean ± SD (*n* = 3), and experiments were performed in triplicate. Different letters within the same line indicate significant differences (*p* < 0.05), as determined by Duncan's multiple range test. During phenotypic analysis, all lines exhibited consistent phenotypes. For clarity and representation, the figure presents data from the representative line *mek1‐2* and *MEK1*‐OE‐#6.

The *mek1* mutants exhibited significantly increased sensitivity to salt stress compared to WT plants, displaying symptoms such as wilting and chlorosis (Figure 4a). These findings suggest that *SlMEK1* may function as a positive regulator of the salt stress response. While HRW application significantly enhanced salt tolerance in WT plants, its promotive effects were markedly weaker in *mek1* mutants, as demonstrated by reduced plant height, fresh weight and dry weight (Figure 4b,d,e). Similar results were seen in root length and leaf area (Figure S9a–d). Thus, *SlMEK* is essential for H_2_‐ehanced salt tolerance.

*SlMEK1* overexpression lines were generated using an *Agrobacterium tumefaciens* ‐mediated transgenic approach. The T2 plants showed highly consistent phenotypic characteristics (Figure S10a). qRT‐PCR analysis indicated that the expression of *SlMEK1* in *MEK1*‐OE‐#1, *MEK1*‐OE‐#3 and *MEK1*‐OE‐#6 lines was increased by 4.45‐, 6.8‐ and 5.65‐fold, respectively (Figure S10b). Accordingly, treatments were applied to three independent lines. To illustrate the representative phenotype, results from the *MEK1*‐OE‐#6 mutant are shown. The *MEK1*‐OE lines exhibited significantly higher salinity tolerance compared to WT (Figure 4f), indicating the positive role of *SlMEK*1 in salt stress tolerance. Compared to WT, HRW alleviated the inhibitory effect of salt stress on plant height, stem diameter, dry weight and fresh weight in *MEK1*‐OE lines (Figure 4g–j). Furthermore, the NaCl‐induced inhibition of total root length and leaf area in *MEK1*‐OE lines was effectively mitigated by HRW (Figure S11a–d). These results further support the essential role of *SlMEK1* in H_2_‐mediated alleviation of salt stress.

Ion homeostasis analysis showed that in WT, salt stress significantly increased Na^+^ accumulation and decreased K^+^ content in tomato seedings compared to the control (Figure S12a,b). HRW effectively mitigated this imbalance by reducing Na^+^ accumulation and maintaining K^+^ stability, resulting in a significantly higher K^+^/Na^+^ ratio (Figure S12a–c). Among the genetic materials, *MEK1*‐overexpressing lines exhibited an ion homeostasis regulation pattern similar to that of WT (Figure S12a–c). In contrast, *mek1* lines under salt stress displayed more severe ion homeostasis disruption, characterised by excessive Na^+^ accumulation, a significant decrease in K^+^ content, and a sharp reduction in the K^+^/Na^+^ ratio (Figure S12a–c). Moreover, the mitigating effect of HRW on these imbalances was markedly weakened in *mek1* lines.

Tolerance index analysis demonstrated that HRW treatment effectively increased the tolerance index in WT plants under salt stress compared to the control (Figure S13). Among the genetic materials, *MEK1*‐OE lines achieved a tolerance index similar to HRW‐treated WT, showing significantly higher values under NaCl treatment (Figure S13a). Conversely, *mek1* lines under salt stress experienced severe growth inhibition, reflected by a notably lower tolerance index (Figure S13a). Moreover, the capacity of HRW to increase the tolerance index was significantly impaired in *mek1* mutants.

### 2.4 *S*‐Nitrosylation of MEK1 at Cys172 Increases Its Activity

**FIGURE 5:**
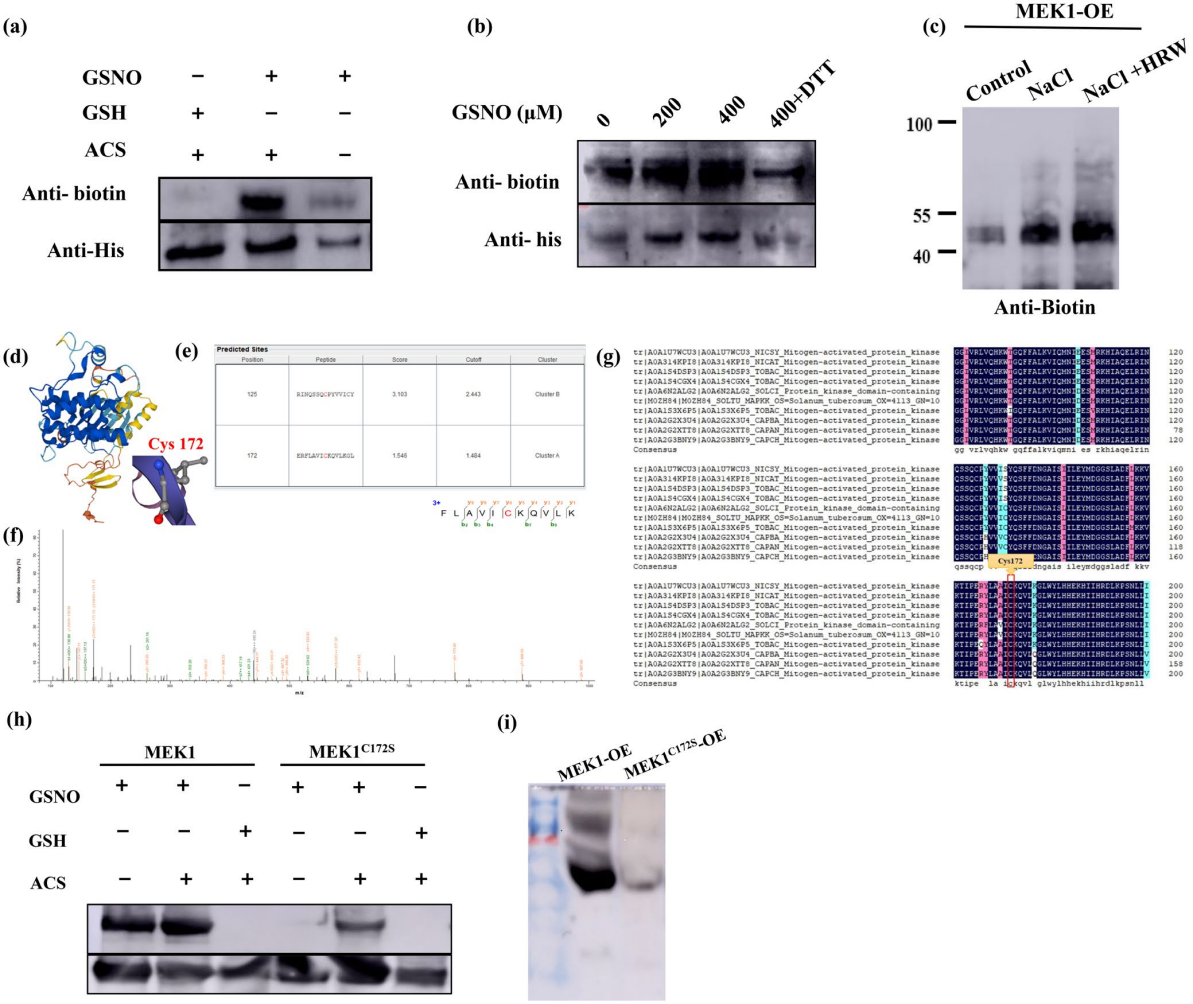
Immunoblot detection of *S*‐nitrosylation. (a) In vitro S‐nitrosylation assay of His‐MEK1 (SNO‐MEK1) treated with GSNO. A sample without sodium ascorbate (Asc) treatment served as a negative control. (b) Immunoblotting analysis of *S*‐nitrosylation of His‐MEK1 (SNO‐MEK1) following treatment with GSNO. Seedlings were sprayed with Mock or 200 to 400 μM GSNO. (c) In vivo S‐nitrosylation assay of MEK1‐HA. Samples without Asc treatment served as negative controls. Four‐week‐old plants were subjected to different treatments for 7 days, and histones were extracted and analysed for specific histone marks. (d) Structural modelling of MEK1. The domain of MEK1 (Uniprot entry O48616) was modelled using the SwissProt Modelling server, and the 3D models were visualised with Swiss‐PdbViewer. (e) *S*‐nitrosylation site prediction using GPS‐SNO software. (f) Mapping of S‐nitrosylation sites in MEK1 protein via liquid chromatography tandem‐mass spectrometry (LC–MS/MS). Analysis of trypsin‐digested and biotin‐charged MEK1 peptides identified Cys‐172 as an *S*‐nitrosylated residue, with b‐ and y‐type productions indicated. (g) Sequence alignment performed using Clustal Omega. Cysteine residues *S*‐nitrosated in closely related species are highlighted in red. Links to proteins with ≥ 90% sequence identity to MEK1 is provided based on UniProt Reference Clusters. (h) In vitro *S*‐nitrosylation assay of His‐MEK1 and His‐MEK1^C172S^. (i) In vivo S‐nitrosylation assay of MEK1 and MEK1^C172S^ in OE‐*MEK1* (MEK1‐HA), OE‐*MEK1* ^C172S^ (MEK1^C172S^‐HA) seedlings. Protein *S*‐nitrosylation levels were quantified and normalised to the loading control.

Protein *S*‐nitrosylation represents a key mechanism by which NO exerts its signal functions (Zhang et al. 2019). To investigate whether H_2_ regulates the *S*‐nitrosylation of MEK1 protein in response to salt stress, we employed the biotin‐switch assay. Our results revealed that GSNO induced *S*‐nitrosylation of the recombinant His‐MEK1 protein (Figure 5a,b). In contrast, treatment with increasing concentrations of GSH yielded only weak protein bands, confirming that the observed signal in GSNO‐treated samples was attributable to NO (Figure 5a). The *S*‐nitrosylation of recombinant His‐MEK1 protein increased in a dose‐dependent manner with higher GSNO concentrations. However, weak protein bands were also detected following treatment with 100 mM dithiothreitol (DTT) (Figure 5b), indicating that MEK1 can undergo *S*‐nitrosylation in vitro. To determine whether MEK1 is *S*‐nitrosylated in vivo, we performed an *S*‐nitrosylation assay using MEK1‐HA seedlings. The *S*‐nitrosylation level of MEK1 increased under NaCl treatment and was further enhanced by the combination of NaCl and HRW (Figure 5c). Therefore, these findings demonstrate that MEK1 undergoes *S*‐nitrosylation both in vitro and in vivo, and this modification plays a role in H_2_‐enhanced salinity tolerance.

The domain of MEK1 (Uniprot entry O48616) was modelled using the SwissProt Modelling server (Figure 5d). Using GPS‐SNO software with the threshold set to ‘High’, we predicted that MEK1 might undergo *S*‐nitrosylation at Cys‐172, and the 3D model of this site was visualised with Swiss‐PdbViewer (Figure 5d,e). To precisely identify the modified Cys residues in *S*‐nitrosylated protein, we employed a site‐specific proteomics approach. Biotinylated peptides were enriched and purified by affinity purification using streptavidin agarose beads and then analysed by LC–MS/MS using an LTQ Orbitrap Elite mass spectrometer. Mass spectrometric analysis of the MEK1 recombinant protein identified Cys‐172 as an *S*‐nitrosylated residue (Figure 5f). Intriguingly, Cys‐172 is highly conserved across species, as demonstrated by protein sequence comparisons of ten closely related species, making it an ideal model for studying MEK1's *S*‐nitrosylation (Figure 5g). Given the structural similarity between serine (Ser or S) and Cys, serine is commonly used as a non‐nitrosylatable substitution (Zhan et al. 2018). To investigate the role of Cys‐172, we replaced it with serine (C172S) to create a mutant MEK1^C172S^‐HIS recombinant protein and OE‐MEK1^C172S^ transgenic plants. Further analysis showed that the *S*‐nitrosylation level of His‐MEK1^C172S^ was significantly lower than that of His‐MEK1 (Figure 5h). This finding indicates that the substitution of Cys‐172 with serine (Ser; MEK1^C172S^) substantially reduced the *S*‐nitrosylation level of His‐MEK1. Consistent with this, the *S*‐nitrosylation of OE‐MEK1^C172S^ was reduced compared to that of OE‐MEK1 in vivo (Figure 5i). Therefore, in vitro and in vivo results suggest that MEK1 undergoes *S‐nitrosylation* at Cys‐172.

### 2.5 *S*‐Nitrosylation of MEK1 Positively Regulates H_2_ ‐Enhanced Salt Tolerance in Tomato Seedlings

**FIGURE 6:**
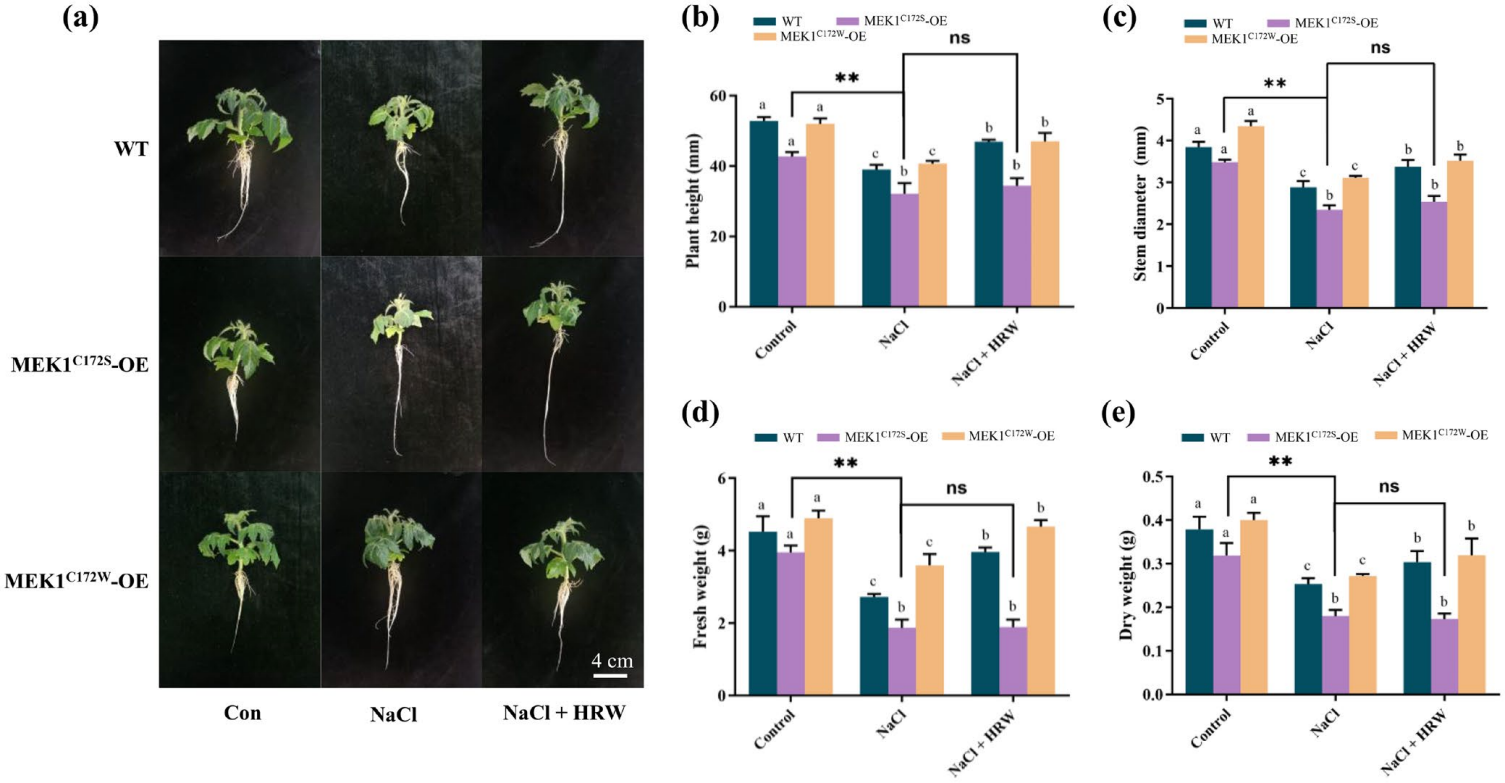
Phenotypic assessment of WT, OE‐MEK1 (MEK1‐HA), OE‐MEK1^C172S^ (MEK1^C172S^‐HA) and OE‐MEK1^C172W^ (MEK1^C172W^‐HA) plants under control, NaCl and NaCl + HRW treatments. (a) plant height, (b) stem diameter, (c) fresh weight and (d) dry weight. Measurements were taken 7 days post‐treatment. Data are expressed as mean ± SD (*n* = 3), and experiments were performed in triplicate. Different letters within the same line indicate significant differences (*p* < 0.05), as determined by Duncan's multiple range test.

To evaluate the function of *S*‐nitrosylated MEK1 in vivo, three overexpression lines (MEK1‐OE, MEK1^C172S^‐OE and MEK1^C172W^‐OE) were generated. MEK1‐OE and MEK1^C172W^‐OE lines exhibited comparable performance in plant height, stem diameter, fresh weight and dry weight (Figure 6a–e). In contrast, these parameters were significantly reduced in the MEK1^C172S^‐OE line compared to the MEK1‐OE and MEK1^C172W^ lines (Figure 6a–e). Notably, MEK1^C172W^‐OE plants displayed enhanced salinity tolerance relative to MEK1‐OE plants, while MEK1^C172S^‐OE lines showed reduced tolerance, as evidenced by phenotypic analyses (Figure 6b–e). HRW improved salt resistance in MEK1‐OE and MEK1^C172W^‐OE lines; however, the promotive effects of HRW on MEK1^C172S^‐OE were less pronounced (Figure 6a–e). These results demonstrate that *S*‐nitrosylation of MEK1 positively contributes to enhanced salt tolerance and plays a critical role in H_2_‐mediated salt tolerance.

Further investigation into the post‐translational modification of MEK revealed that MEK1^C172W^‐HA lines, akin to *MEK1*‐overexpression lines, inherently enhanced ion homeostasis regulation. These lines maintained lower Na^+^ and higher K^+^ levels under salt stress, thereby resulting in a high K^+^/Na^+^ ratio (Figure S12a–c). Conversely, MEK1^C172S^‐HA lines exhibited ion homeostasis disruption similar to *mek1* lines, characterised by exacerbated Na^+^ influx and K^+^ loss, and a low K^+^/Na^+^ ratio. Additionally, these lines showed significantly reduced improvement in ion homeostasis in response to HRW (Figure S12a–c). These findings highlight that *S*‐nitrosylation at Cys‐172 is essential for the H_2_‐dependent regulation of ion homeostasis and the maintenance of a protective K^+^/Na^+^ ratio under saline conditions.

Additionally, MEK1^C172W^‐HA lines inherently possessed enhanced stress tolerance, maintaining higher tolerance index under salt stress (Figure S13b). In contrast, MEK1^C172S^‐HA lines showed significantly reduced improvement in tolerance index in response to HRW (Figure S13b). Therefore, *S*‐nitrosylation at the Cys172 site of MEK1 is essential for the H_2_‐mediated enhancement of salt tolerance.

### 2.6 *S*‐Nitrosylation of MEK1 at Cys‐172 Enhances Its Interaction With GSNOR

**FIGURE 7:**
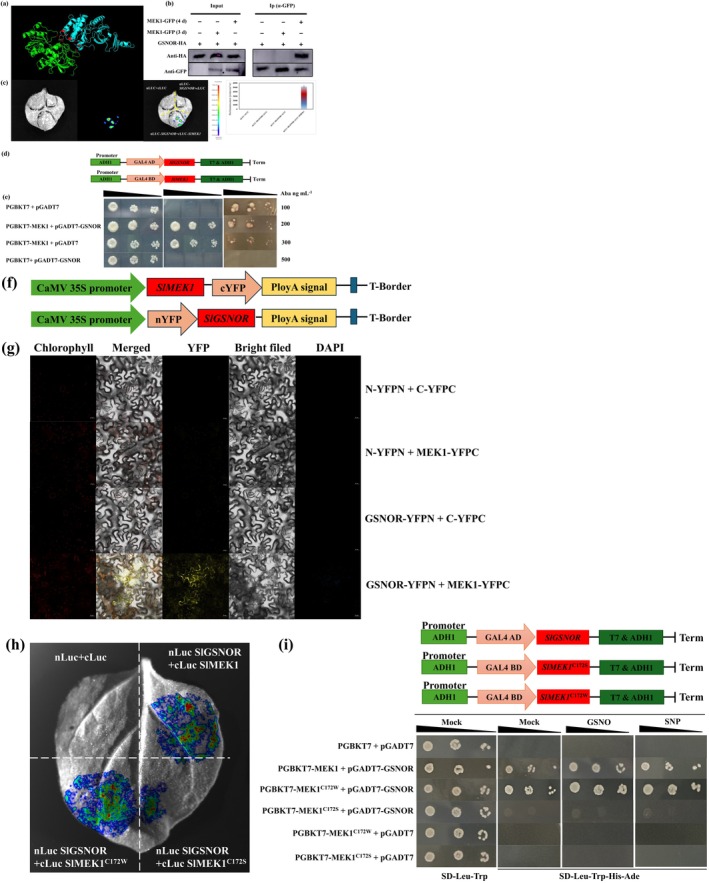
*S*‐nitrosylation of MEK1 at Cys‐172 enhances its interaction with GSNOR. (a) Protein interaction model of MEK1 and GSNOR. (b) Co‐IP assay showing MEK1 and GSNOR interaction. MEK1 and GSNOR were tagged with GFP and HA, respectively, and the precipitate was detected using anti‐GFP and anti‐HA antibodies. (c) Biomolecular luciferase complementation (LUC) assay showing the interaction between MEK1 and GSNOR. Relative quantification was performed using ImageJ software. (d) Schematic structure of the yeast two‐hybrid (Y2H) vector. (e) Y2H assay confirming the interaction between MEK1 and GSNOR. (f) Schematic structure of the bimolecular fluorescence complementation (BiFC) vector. (g) BiFC assay showing the interaction between MEK1 and GSNOR in tobacco epidermal leaves. Scale bar, 20 μm. (h) LUC assay demonstrating the interaction of MEK1^C172S^ and MEK1^C172W^ with GSNOR. (i) Y2H assay was carried out with cells co‐transformed with the indicated constructs and grown on selective media supplemented with the corresponding concentrations of GSNO and SNP to assess yeast growth.

*S*‐nitrosylation, regulated by GSNOR, is a pivotal mechanism for NO‐modulated stress tolerance in plants (Gong et al. 2019). Our results indicate the involvement of NO and MEK1 *S*‐nitrosylation in H_2_‐enhanced salt tolerance in tomato seedlings. To further investigate the molecular mechanism underlying H_2_‐mediated MEK1‐enhanced salt tolerance, we hypothesized an interaction between SlMEK1 and SlGSNOR. Using an online protein interaction prediction tool, we generated a model of SlMEK1 and SlGSNOR interaction (Figure 7a). Co‐IP assays confirmed the interaction between MEK1 and GSNOR in tobacco leaves after 4 days of expression (Figure 7b). Subsequently, a LUC assay revealed that co‐expression of nLUC‐GSNOR and cLUC‐MEK1 in tobacco leaves resulted in biofluorescence, with significantly higher intensity than the empty vector control, indicating an in vivo interaction between GSNOR and MEK1 (Figure 7c). Additionally, the CDS of GSNOR and MEK1 were cloned into pGADT7 and pGBKT7 plasmids, respectively and transformed into yeast cells for further validation (Figure 7d). Since the fusion of SlMEK1 to the pGBKT7 plasmid showed self‐activation, the optimum concentration of AbA (300 ng mL^−1^) was screened to suppress this self‐activation (Figure 7e). Interestingly, yeast cells co‐transformed with MEK1‐BD and GSNOR‐AD grew well in a deficiency medium supplied with AbA (300 ng mL^−1^; Figure 7f). To further validate Y2H results, the CDS of SlMEK1 and SlGSNOR were fused to cYFP and nYFP, respectively (Figure 7f). YFP fluorescence was exclusively observed in the co‐expression of MEK1 and GSNOR, localised to the cell membrane and cytoplasm (Figure 7g). These findings confirm that the interaction between MEK1 and GSNOR occurs in vivo and in vitro.

To investigate whether the interaction between MEK1 and GSNOR is influenced by protein *S*‐nitrosylation, we mutated the Cys 172 modification site of MEK1. Luc analysis revealed that the biological fluorescence intensity was significantly lower in tobacco leaves co‐expressing MEK1^C115S^ and GSNOR compared to the co‐expression MEK1 and GSNOR (Figure 7h). Conversely, co‐expression of MEK1^C115W^ and GSNOR resulted in a markedly higher fluorescence intensity relative to MEK1 and GSNOR co‐expression. Additionally, fusion of MEK1^C115S^ into the pGBKT7 vector and co‐transformation with AD‐GSNOR revealed that yeast cells failed to grow in the deficient medium (Figure 7i). Similarly, co‐transformation of BD‐MEK1^C115W^ and AD‐GSNOR into yeast produced results consistent with those obtained from the Luc analysis (Figure 7i). Furthermore, the growth of yeast strain co‐transformed with BD‐MEK1 or BD‐MEK1^C115W^ and AD‐GSNOR was significantly promoted by different NO donors (SNP and GSNO), whereas the co‐transformant with BD‐MEK1^C115S^ and AD‐GSNOR showed no response to these donors (Figure 7i). Therefore, these findings suggest that *S*‐nitrosylation of MEK1 strengthens its interaction with GSNOR.

### 2.7 H_2_ Enhances the Interaction Between GSNOR and *S*‐Nitrosylated MEK1 Under Salt Stress

**FIGURE 8:**
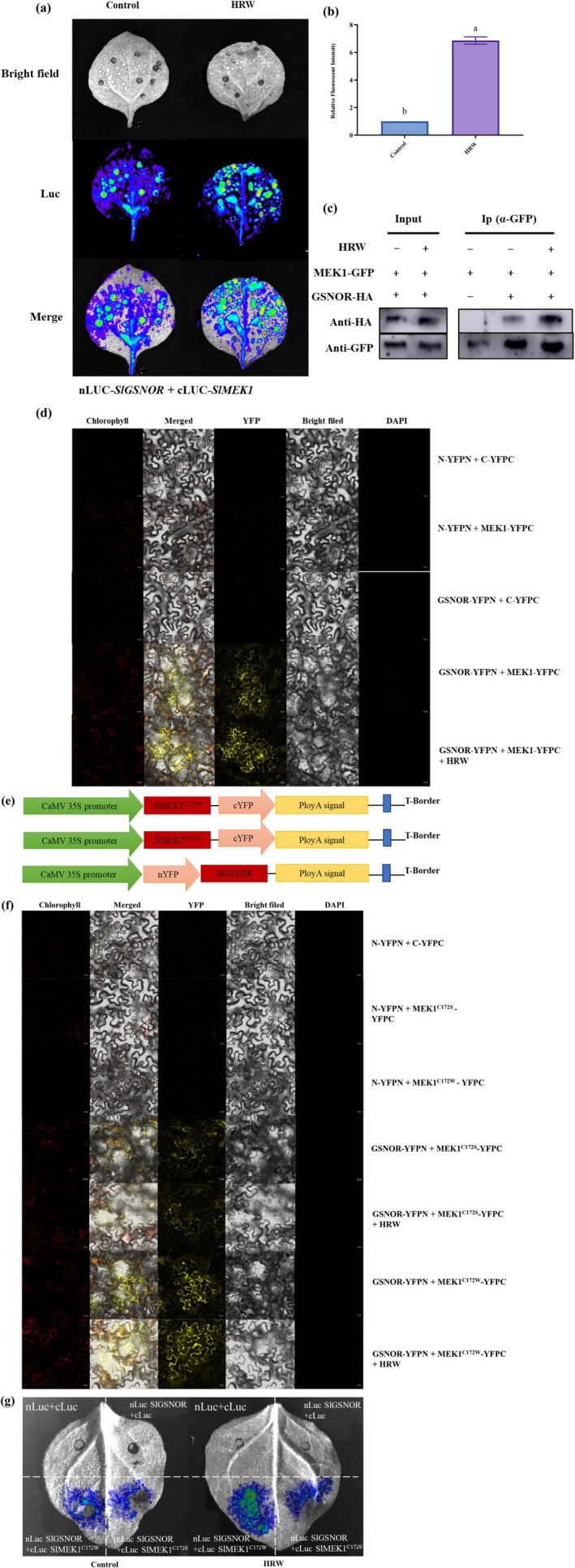
H_2_ enhances the interaction between GSNOR and *S*‐nitrosylated MEK1 under salt stress. (a, b) Luciferase complementation assay (Luc) demonstrating that HRW regulates the interaction between MEK1 and GSNOR. Relative quantification was performed using ImageJ software. (c) Co‐Immunoprecipitation (Co‐IP) confirming that HRW regulates the interaction between MEK1 and GSNOR. (d) Bimolecular fluorescence complementation (BiFC) assay validating HRW‐regulated interactions between MEK1 and GSNOR. (e) Schematic representation of the BiFC constructs MEK1^C172S^‐YFPC and MEK1^C172W^‐YFPC. (f) BiFC assay validating HRW‐regulated *S*‐nitrosylation of MEK1 protein at the Cys 172 site and its interaction with GSNOR. (g) LUC demonstrating HRW‐regulated *S*‐nitrosylation of MEK1 at the Cys 172 site and its interaction with GSNOR.

The subcellular localization of MEK1 was determined in *vivo* using translational fusions to GFP expressed by the CaMV 35S‐SlMEK1‐GFP constructs. Confocal microscopy of *N*. *benthamiana* leaves transiently expressing these constructs showed that SlMEK1‐GFP localised to the plasma membrane and cytoplasmic compartments (Figure S14). Notably, HRW treatment slightly enhanced the green fluorescence intensity at the plasma membrane compared to the untreated control (Figure S14). Further analysis revealed that mutation at Cys172 did not alter the subcellular localization of MEK1 (Figure S15), indicating that *S*‐nitrosylation does not affect MEK1 cellular compartmentalization. To investigate the influence of H_2_ on the interaction between GSNOR and MEK1, we performed a Luc assay. Constructs containing nLUC‐GSNOR and cLUC‐MEK1 were infiltrated into tobacco leaves, followed by irrigation with HRW. HRW treatment resulted in a stronger luminescence signal in the SlGSNOR‐nLuc/cLuc‐SlMEK1 co‐expressing region (Figure 8a). Additionally, the relative fluorescence intensity was significantly higher in HRW‐treated samples compared to the control (Figure 8b). To validate these observations, we conducted a Co‐IP assay by overexpressing MEK1‐GFP and GSNOR‐HA in HRW‐treated tobacco leaves. GSNOR‐HA was detected in MEK1‐GFP immunoprecipitates, and the signal was enhanced by HRW treatment (Figure 8c), indicating that H_2_ strengthens the interaction. This finding was further corroborated by BiFC assays, which showed increased YFP fluorescence in HRW‐treated tobacco leaves (Figure 8d).

To investigate whether H_2_ regulates the interactions between GSNOR and *S*‐nitrosylated MEK1, we performed BiFC assays. MEK1^C172S^ and GSNOR were cloned into YFPC and YFPN, respectively, and these constructs were co‐transfected into tobacco leaves (Figure 8e). Weak YFP signals were observed in the cell membrane and cytoplasm (Figure 8f). In contrast, stronger YFP fluorescence was detected in tobacco leaves co‐expressing MEK1^C172W^‐YFPC and GSNOR‐YFPN (Figure 8f). The MEK1^C172W^‐YFPC and GSNOR‐YFPN constructs were then co‐infiltrated into tobacco leaves, which were treated with HRW. Following HRW treatment, a stronger and more distinct YFP fluorescence signal was observed in the cell membrane (Figure 8f). In contrast, the opposite effect was observed in leaves expressing MEK1^C172S^‐YFPC (Figure 8f). HRW treatment led to a stronger luminescence signal in regions co‐expressing SlGSNOR‐nLuc/cLuc‐SlMEK1^C172W^, whereas only weak signals were detected in leaves co‐expressing SlGSNOR‐nLuc/cLuc‐MEK1^C172S^, with no enhancement observed after HRW treatment (Figure 8g). These results suggest that H_2_ enhances the interaction between MEK1 and GSNOR through *S*‐nitrosylation at the Cys172 site of MEK1.

## 3 Discussion

Salinity stress reduces plant growth and productivity, while plants have evolved complex signalling pathways to counteract it. H_2_, known for its non‐toxic, safe and efficient properties, has been widely studied for its role in plant growth and stress responses (Ohta 2011). Exogenous HRW application mitigates salt stress‐induced growth inhibition in rice (Xu et al. 2013), and our show that 75% HRW optimally alleviates NaCl‐induced growth suppression in tomato seedlings (Figure S1). This align with previous work showing that HRW enhances salt tolerance in tomato roots by upregulating strigolactone (SL) biosynthetic and signalling genes (Ye et al. 2025). Additionally, HRW's function may depend on the NO signalling pathway (Zhao et al. 2021), as evidenced by H_2_‐supplying AB@hMSN nanoparticles inducing tomato lateral root formation in a NO‐dependent manner (Wang et al. 2020). To explore HRW's role in salt stress alleviation via NO, we used exogenous GSNO (NO donor) and cPTIO (NO scavenger) (Figure 1). HRW combined with GSNO significantly alleviates salt stress, while cPTIO diminished HRW's mitigating effect, indicating NO is required for HRW‐mediated salt stress alleviation and suggesting synergistic H_2_‐NO signalling. However, progress in deciphering this interaction is hindered by the scarcity of specific molecular tools. While DCPIP has been employed as a putative H_2_ biosynthesis inhibitor (Su, Nie, et al. 2019), its specificity and reliability require validation. Developing highly specific inhibitors and systematically applying them to investigate gaseous signal crosstalk is a crucial for elucidating H_2_‐NO synergistic mechanism.

S‐nitrosoglutathione reductase (GSNOR) plays a crucial role in maintaining the homeostasis of GSNO and NO within cells (Jahnová et al. 2019). GSNOR knockout elevates endogenous NO levels, altering stress responses (Zhang et al. 2022; Li et al. 2025), as seen in *gsnor1/hot5/par2* mutants with disrupted NO homeostasis and altered responses to bacterial pathogens (Yun et al. 2011). Wei et al. (2025) demonstrated that *GSNOR* overexpression (*GSNOR*‐OE) reduces NO and *S*‐nitrosothiols under saline‐alkali stress, enhancing tomato tolerance, while *GSNOR*‐RNAi increases NO, exacerbating stress. Despite lowering NO, GSNOR‐OE promotes saline‐alkali tolerance. Our study revealed that *SlGSNOR*‐OE lines exhibit decreased salt tolerance, and HRW treatment fails to alleviate stress, but GSNO rescues this effect (Figure 2), supporting H_2_‐mediated adaptation via NO signalling. NO's dual role—signalling at low levels but causing damage at high levels—explains this paradox. Liu, Cao, et al. (2024) highlighted NO's regulation of GABA‐TP via *S*‐nitrosylation, emphasising NO balance for stress adaptation. Therefore, GSNOR modulates NO levels, preserving its signalling benefits while preventing toxicity, acting as a positive regulator of saline‐alkali tolerance.

Previous research indicates that stress conditions elevate intracellular NO levels, enhancing broad‐spectrum stress tolerance in plants (Fancy et al. 2017). In our study, NO/SNO emission/accumulation increases under salt stress, further boosted by H_2_ treatment, improving salt tolerance (Figure 1). While NO is vital for plant physiology, excessive NO or reactive nitrogen species (RNS) can cause cellular toxicity, ultimately triggering programmed cell death or necrotic cellular demise (Huang et al. 2024). Thus, maintaining NO homeostasis is crucial for preserving its signalling role in horticultural products. GSNOR, the key enzyme regulating cellular levels of NO and SNO, ensures NO/SNO homeostasis. Loss of GSNOR function increases NO and SNO levels (Jahnová et al. 2019). For example, heat stress (38°C for 4 h) reduces GSNOR activity in sunflower (*Helianthus annuus*) hypocotyls, decreasing protein abundance and *GSNOR* gene expression while increasing SNO accumulation (Chaki 2007). Elevated NO and SNO levels lead to GSNOR deficiency (Figure 3), disrupting *S*‐nitrosylation balance. Furthermore, NO and SNO levels are negatively correlated with GSNOR activity (Figure 3), consistent with heat stress significantly reducing GSNOR activity in sunflower seedlings (Tanou et al. 2009). Moreover, H_2_ treatment enhances GSNOR activity under salt stress, indicating that H_2_ indirectly regulates GSNOR to maintain SNO homeostasis. NO modulates protein *S*‐nitrosylation, while GSNOR catalyses de‐nitrosylation (Malik et al. 2011). Increased RNS levels may enhance proteome‐wide *S*‐nitrosylation, influencing salt stress tolerance in tomato plants. Moreover, exogenous substances like brassinosteroids (BR) also affect *S*‐nitrosylation; BR enhances NO‐mediated *S*‐nitrosylation in salt‐stressed tomato, with GSNO+BR co‐treatment increasing *S*‐nitrosylated protein levels (Wei et al. 2024). In our study, higher *S*‐nitrosylation levels were observed in NaCl and NaCl + HRW treatments compared to the control (Figure 3), indicating that salinity stress and H_2_‐enhanced salinity tolerance promote protein *S*‐nitrosylation. These findings suggest that H_2_ modulates stress responses by regulating *S*‐nitrosylation, likely through elevating endogenous NO levels. Nevertheless, the precise molecular mechanisms of H_2_'s influence on cellular signalling pathways require further investigation.

Previous research by our group revealed that exogenous NO enhances salt tolerance in tomato seedlings by modulating *S*‐nitrosylation levels (Wang et al. 2022). To elucidate the molecular mechanism of H_2_ in salt stress response, we conducted the first comprehensive analysis of *S*‐nitrosylated proteins in tomato under salinity and H_2_‐enhanced salinity tolerance processes. Our profiling revealed complex regulatory networks involved in stress adaptation, with *S*‐nitrosylated proteins primarily implicated in glycerophospholipid metabolism, MAPK signalling, arginine and proline metabolism, nucleotide excision repair and mismatch repair, with distinct patterns in NaCl versus NaCl + HRW treatments (Figure 3). Proteomic analysis demonstrated that salt stress disrupted basal *S*‐nitrosylation homeostasis, as NaCl treatment lacked unique *S*‐nitrosylated proteins compared to the control (Figure S5h). H_2_ restores homeostasis through NO‐dependent thiol redox buffering, likely by modulating GSNOR activity, consistent with the established role of GSNOR‐mediated *S*‐nitrosylation in plant stress resistance (Gong and Shi 2019; Gong et al. 2019). GO enrichment analysis demonstrated that salt stress primarily activated processes related to stress response, metabolic reprogramming and protein folding, whereas HRW treatment shifted the cellular state toward homeostasis recovery and energy regulation (Figure S6). Notably, HRW specifically enriched functions such as ‘ATP binding’ and ‘purine ribonucleotide binding’, suggesting that maintaining cellular energy status constitutes a key mechanism of HRW‐induced stress mitigation (Figure S6). This regulatory pattern is consistent with findings from a study on *S*‐nitrosylation under saline‐alkaline stress, which identified 282 common *S*‐nitrosylated proteins significantly enriched in processes such as ‘protein folding’ and ‘proton transmembrane transport’, as well as cellular components including ‘ribosome,’ ‘mitochondrial inner membrane’ and ‘ATPase complex’ (Wei et al. 2025). Therefore, the tomato response to Na^+^ stress involves the rapid regulation of key proteins—such as folding chaperones, proton pumps and photosynthetic/translational components—via *S*‐nitrosylation. HRW likely enhances salt tolerance by influencing this modification network, particularly at nodes related to energy and protein homeostasis. This restoration highlights H_2_'s ability to re‐establish modification homeostasis via NO‐mediated redox control. Our earlier LC–MS/MS proteomic profiling identified 1054 *S*‐nitrosylated proteins implicated in NO‐mediated salt stress mitigation in tomato, including MAPK pathway components (Wei et al. 2022). The discovery of 8 MAPK cascade proteins undergoing *S*‐nitrosylation represents a paradigm shift, as MAPK activation is typically phosphorylation‐dependent (Nakagami et al. 2005). Furthermore, 735 share *S*‐nitrosylated targets between HRW and GSNO treatments (38.2% overlap) establish a functional link between H_2_ and NO signalling (Figure 3, Table S1). These findings highlight critical targets for H_2_‐enhanced salt tolerance. These findings not only emphasise the functional crosstalk between H_2_ and NO signalling, but also uncover a novel regulatory mechanism by which gaseous molecules modulate MAPK cascades. Notably, the identification of 15 MAPK pathway components within this overlapping subset points to conserved redox regulation points across gaseous signalling pathways, providing new insights into the molecular basis of stress tolerance in plants.

The MAPK signalling pathway is central to plant stress responses, with members like Arabidopsis MKK2 and MKK9 showing divergent roles in salt stress adaptation—MKK2 enhances tolerance, while MKK9 acts as a negative regulator (Teige et al. 2004; Xu et al. 2008). This highlights the functional divergence and complexity of MAPK cascade signalling. Our findings demonstrate that *SlMEK1* is critical for salt tolerance in tomato, as its mutation impairs seedling development under stress, while overexpression improves tolerance (Figure 4). Increasing evidence suggests an intricate interplay between MAPK signalling pathways and NO signalling (Lv et al. 2017; Ye et al. 2013), with spatiotemporal specificity. For instance, under salt stress, the expression peaks of *CsPLDα* and *CsNMAPK* in cucumber occur at 1 and 3 h post‐treatment, respectively, while NO accumulation peaks as early as 1 h. This temporal discrepancy suggests that NO likely functions as an upstream signalling molecule activating the MAPK cascade (Ji et al. 2017). Our study shows that H_2_‐triggered NO accumulation fails to rescue the salt‐sensitive phenotype of *mek1* mutants. However, HRW‐triggered NO accumulation significantly enhances salt tolerance in *SlMEK1*‐overexpressing seedlings (Figure 4). This differential regulatory effect indicates that *SlMEK1* is a key component in H_2_‐mediated salt tolerance, and its functional absence may completely block downstream signalling pathways. Furthermore, *SlMEK1* appears to function downstream of the H_2_‐NO signalling module, which predominantly exerts its effects through the activation of *SlMEK1*.

The MAPK cascade is a key signalling pathway that transmits stress signals through phosphorylation and dephosphorylation, triggering stress resistance responses. In medical research, *S*‐nitrosylation of ERK inhibits its phosphorylation and induces tumour cell apoptosis (Feng, Sun, et al. 2013). In plants, stress‐induced NO accumulation activates the MAPK pathway, likely via ROS modulation (Lv et al. 2017; Ye et al. 2013). While MAPK may regulate NO production, NO may activate the MAPK cascade to propagate stress signals. However, *S*‐nitrosylation of MAPK kinases in this process remains unexplored. To elucidate the H_2_‐NO‐MEK1 signalling pathway, we examined MEK1's *S*‐nitrosylation status and found it undergoes *S*‐nitrosylation modification both in vitro and in vivo (Figure 5), highlighting its role in H_2_‐mediated salt tolerance. Cysteine (Cys) oxidation acts as a redox sensor, facilitating environmental perception defence responses (Spadaro et al. 2010; Diaz‐Vivancos et al. 2015; Sevilla et al. 2015; Begara‐Morales et al. 2016). Mass spectrometry identified Cys172 as the *S*‐nitrosylation site on MEK1 (Figure 5). Notably, Cys172 of MEK1 is conserved across ten phylogenetically related species, highlighting its high evolutionary conservation. A denitrosylation assay yields a faint signal, suggesting that Cys172 is not the sole site (Hu et al. 2017; Zhan et al. 2018). However, Cys172 remains a critical *S*‐nitrosylation site governing MEK1 activity. Analogous studies show *S*‐nitrosation of ascorbate peroxidase (APX) at Cys32 regulates oxidative and salt stress response (Begara‐Morales et al. 2014; Yang et al. 2015), and *S*‐nitrosation of GSNOR1 at Cys10 promotes its degradation via selective autophagy, enhancing seed germination and hypoxia stress tolerance (Zhan et al. 2018). We mapped H_2_‐dependent salt tolerance to *S*‐nitrosylation of SlMEK1 at Cys172. Specifically, the nitrosomimetic C172W variant exhibits enhanced stress resilience, whereas the de‐nitrosylation C172S mutant is unresponsive to H_2_ (Figure 6). Plants adapt to saline‐alkaline environments through Na^+^ recycling via preferential transporters, preventing excessive Na^+^ accumulation (Zhang, Li, and Zhu 2018; Yang and Guo 2018). MEK overexpression improves ion balance, mimicking H_2_ effects, while MEK knockout lines lost ion homeostasis and H_2_ responsiveness (Figure S12). The MEK1^C172W^ conferred constitutive ion homeostasis under salt stress, whereas the MEK1^C172S^ impaired both basal homeostasis and H_2_ protection (Figure S12). Together, these findings demonstrate that the H_2_‐MEK1 module orchestrates plant salt tolerance through *S*‐nitrosylation‐mediated molecular pathways, highlighting its evolutionary and functional significance.

Deciphering gasotransmitter signalling networks remains a critical challenge. Our study reveals that H_2_ elevates endogenous NO levels and upregulates MEK1 expression (transcriptional and translational) in tomato seedlings. Genetic evidence highlights the pivotal role of MEK1 in H_2_‐enhanced salt tolerance, although its precise mechanistic action warrants further investigation. Importantly, we identify NO as the key signalling molecule mediating H_2_‐induced MEK1 activation, with GSNOR emerging as the master regulator of NO homeostasis (Feng, Wang, et al. 2013). *S*‐nitrosylation, regulated by GSNOR, is considered an important pathway for NO bioactivity (Gong et al. 2019). We therefore speculate that plants may be regulated by the crosstalk module H_2_‐NO‐MAPK in response to adversity conditions. We demonstrate that MEK1 interacts with GSNOR in vivo and in *vitro* (Figure 7). Zhou et al. (2016) revealed that AtCaM4‐GSNOR interactions reduce GSNOR activity, promoting NO accumulation and salt resistance in Arabidopsis. Similarly, ZjMAPKK4 interacts with ZjNAC78 to regulate downstream *ZjICE‐ZjCBF* genes, thereby modulating cold tolerance in jujube (Wang et al. 2025). These findings highlight protein interactions as a key mechanism in plant responses to stress. Furthermore, we observed that HRW addition enhances the MEK1‐GSNOR interaction (Figure 8), indicating that H_2_ promotes this interaction via NO accumulation. OsNAC29 interacts with OsRACK1A and OsMAPK3/6 to form an immune complex, where OsMAPK3 phosphorylation prevents OsNAC29 degradation, enhancing rice blast resistance (Lu et al. 2024). Additionally, Yan et al. (2025) demonstrated that mitogen‐activated protein kinase kinase kinase5 (OsMAPKKK5) phosphorylates brassinosteroid‐signalling kinase1‐1 (OsBSK1‐1), strengthening their interaction and regulating rice yield traits. These findings highlight the importance of post‐translational modifications in protein interactions. Consistent with this, our results reveal that the interaction between MEK1 and GSNOR is diminished when MEK1 fails to undergo *S*‐nitrosylation, and the enhancing effect of H_2_ on their interaction is also abolished (Figures 7 and 8). Conversely, HRW potentiates the MEK1^C172W^‐GSNOR interaction, evidenced by enhanced membrane YFP signals (Figure 8), indicating that MEK1 *S*‐nitrosylation acts as a key switch for H_2_‐mediated regulation of its interaction with GSNOR. Supporting this, Liu, Wei, et al. (2024) demonstrated that *S*‐nitrosylation of SlP5CR at the Cys‐5 site enhances its NAD(P)H interactions and enzymatic activity, enhancing salt tolerance in tomato. Furthermore, NO‐induced *S*‐nitrosylation of TRI1 at Cys‐140 promotes the TIR1‐Aux/IAA interaction and accelerates Aux/IAA degradation in Arabidopsis. Collectively, these findings highlight protein *S*‐nitrosylation's critical role in modulating protein stability and interactions, enabling plants to adapt to adverse conditions by altering physiological functions.

**FIGURE 9:**
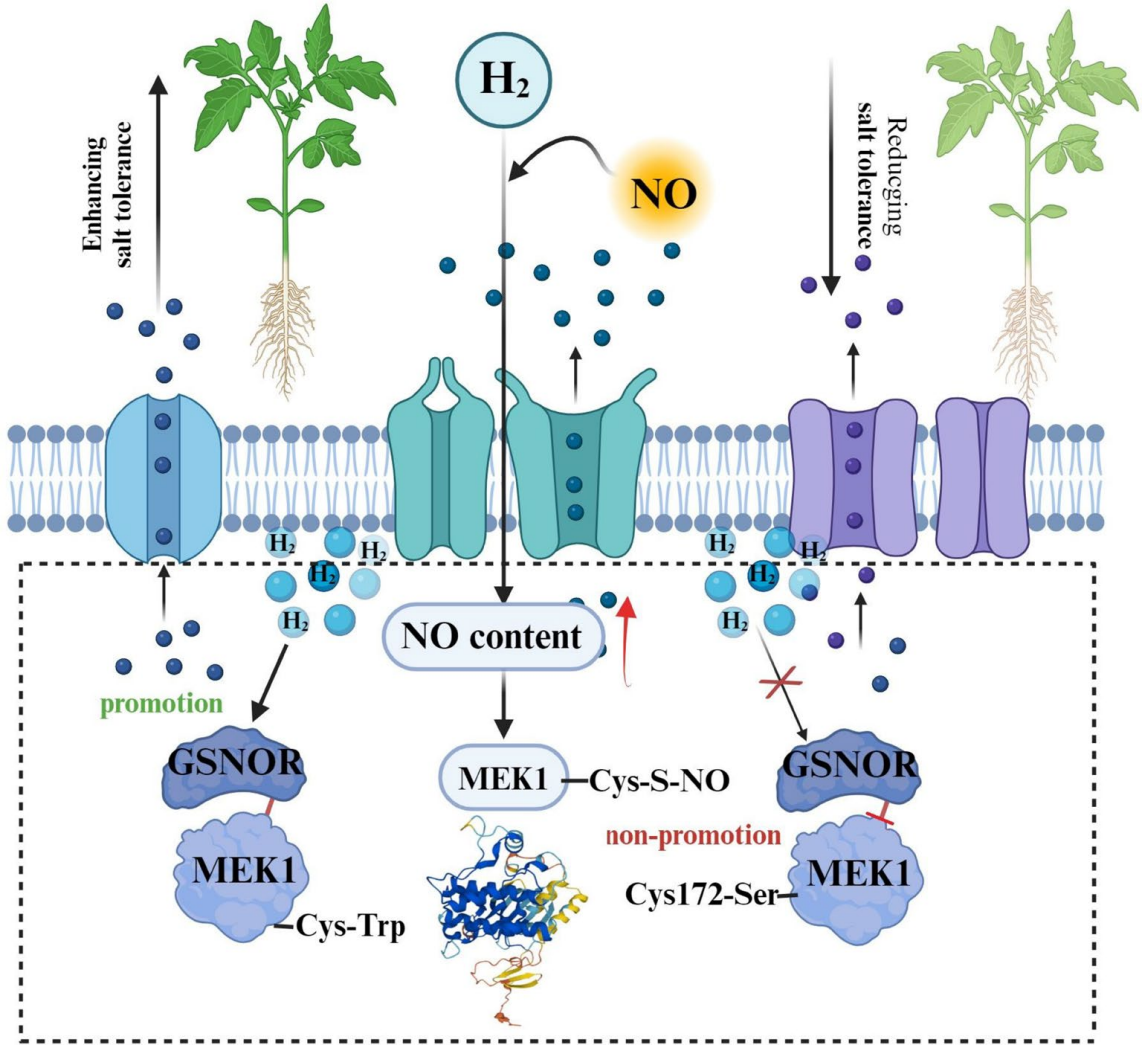
Mechanism of H_2_‐induced MEK1 *S*‐nitrosylation in response to salt stress. Exogenous H_2_ triggered an elevation in endogenous NO level under salt stress, thereby enhancing the enrichment of *S*‐nitrosylated proteins. MEK1 was induced and *S*‐nitrosylated by NO and H_2_ under salt stress. MEK1 underwent *S*‐nitrosylation at Cys 172, which positively contributed to both intrinsic and H_2_‐facilitated salt tolerance. Furthermore, *S*‐nitrosylation of MEK1 increased its interaction with GSNOR, and H_2_ further promoted this interaction under salt stress. Taken together, H_2_ enhances salt tolerance in tomato seedlings by regulating *S*‐nitrosylation of MEK1 and the interaction between MEK1 and GSNOR proteins.

In conclusion, our study establishes a molecular mechanism by which H_2_‐induced NO triggers protein *S*‐nitrosylation under salt stress. MEK1 emerges as a crucial component in H_2_‐mediated salt stress mitigation, with H_2_‐triggered *S*‐nitrosylation of MEK1 representing a previously unrecognised regulatory mechanism for enhancing salt tolerance. This finding advances our understanding of gaseous signalling in plant stress adaptation. Furthermore, we reveal a novel mechanism of GSNOR‐MEK crosstalk, where their H_2_‐dependent interaction is mediated by *S*‐nitrosylation of MEK1 (Figure 9). We propose that *S*‐nitrosylation of MEK1 may modulate its phosphorylation status, thereby regulating its function and the phosphorylation/dephosphorylation of downstream response regulators. This mechanism plays a pivotal role in mitigating adverse stress, offering a promising avenue for future research. The coordinating role of H_2_ in stress adaptation highlights its potential importance in enabling plants to cope with changing environmental conditions. This discovery provides a promising foundation for mitigating the negative impacts of climate change on plant productivity.

## 4 Materials and Methods

### 4.1 Plant Materials and Growth Conditions

Tomato ( *Solanum lycopersicum* L. ‘Micro‐Tom’) was purchased from Plenty Co. Ltd. (Nanjing, China). Seed sterilisation was performed using 1% sodium hypochlorite and thoroughly rinsed with distilled water. Germination was carried out at 28°C under humid conditions. Germinated seedlings were transplanted into a substrate mixture (charcoal: vermiculite = 3:1) and grown for 7 days. After the first true leaves emerged, seedlings were transferred to 1/2 Hoagland nutrient solution for 7 days, followed by full‐strength Hoagland solution for 21 days, until plants reached the three‐leaf and one‐heart stage for subsequent experimental treatments. Plants were maintained in a growth chamber set at 26°C with 16 h of light (12 000 lx), 20°C with 8 h of darkness, and 60% relative humidity.

### 4.2 Constructs and Plant Transformation

To generate knockout mutant lines, sgRNAs containing 20‐bp sequences targeting the exon of *SlMEK1* were designed and inserted into the Cas9 vector. The cassette was subcloned into the PHEE401 vector and transformed into tomato cv. Micro‐Tom via *Agrobacterium tumefaciens* ‐mediated cotyledon tissue culture. Genomic DNA was extracted from the leaves of transgenic lines using a DNA extraction kit (Qiagen, Hilden, Germany). DNA fragments containing the target sites were amplified using specific primers, and the PCR products were sequenced to identify positive lines. The primers used are listed in Table S2.

To generate overexpression lines, the *SlMEK1* gene was cloned into the PAC004‐HA vector and introduced into tomato cv. Micro‐Tom via *Agrobacterium*‐mediated transformation of cotyledon tissue. The primers used are shown in Table S2. Overexpression mutants were confirmed by Western blot (WB).

### 4.3 Real‐Time Quantitative Reverse Transcription PCR (RT‐qPCR)

Real‐time quantitative reverse transcription PCR (RT‐qPCR) was performed to analyse the expression levels of the target gene in knockout and overexpression mutants obtained through genetic transformation. Total RNA was extracted from tomato leaves using the TRIzol reagent (Vazyme, Nanjing, China). qRT‐PCR was performed on the Light Cycler 96 Real‐Time PCR System using the SYBR Green Premix Pro Taq HS qPCR Kit (Accurate Biotechnology), following the manufacturer's protocol. The primers for qRT‐PCR were designed using Primer Premier v5.0 (PREMIER Biosoft, CA, USA) and are listed in Table S3. The relative expression levels of target genes were calculated using the 2‐ΔΔCt method, with ACTIN (NC_015447) as the internal reference. Each experiment included three biological replicates to ensure reproducibility.

### 4.4 Treatments

Purified H_2_ (99.99%, v/v) was generated using an H_2_‐producing apparatus (QL‐300, Saikesaisi Hydrogen Energy Co. Ltd., China) and bubbled into 2 L of Hoagland nutrient solution (20°C) at a rate of 5 mL s^−1^ for 1 h to prepare hydrogen‐rich water (HRW) as a H_2_ donor. The H_2_ concentration in freshly prepared HRW was 0.47 mM, as measured by a dissolved hydrogen portable meter (Trustlex Co. Ltd., ENH‐1000, Japan). The H_2_ content in HRW remained relatively stable for at least 12 h at 20°C (Zhu et al. 2016). This experimental treatment was divided into two parts, with Hoagland nutrient solution serving as the control. First, saturated HRW (100% HRW) was diluted to the required concentrations (10%, 25%, 50% and 75% [v/v]) and combined with NaCl (150 mM). Second, the following treatments were applied for 7 d: NaCl (150 mM), NaCl + HRW (75%), NaCl + GSNO (10 μM, a NO donor), NaCl + HRW (75%) + GSNO (10 μM) and NaCl + HRW (75%) + cPTIO (10 μM, a NO scavenger). These concentrations were optimised in preliminary experiments. Fresh solutions were prepared daily to maintain consistency. To prevent the volatilization of H_2_ and NO, the bottle mouth was tightly sealed with a sealing film. Each treatment was performed in triplicate. Samples were mixed, wrapped in clean tin foil, frozen in liquid nitrogen and stored at −80°C to maintain integrity for subsequent analysis.

### 4.5 Measurement of Morphological Parameters

Tomato seedlings were treated for 7 days and then placed on a black background for vertical photography using a camera (Canon 750D, Japan). The roots were washed, drained and laid flat for scanning with a root scanner (STD4800, Canada). Root morphology was analysed for individual plants using Win RHIZO 5.0 software (Regent Instruments Inc., Quebec City, Canada). Plant height was measured as the distance from the base of the seedling to the growth point using a vernier calliper. Six plants per replicate were measured, and the average value was calculated. Stem diameter was measured using a vernier calliper, with the average value derived from 6 plants per replicate. The total leaf area of each plant was determined using a leaf scanner (YMJ‐C, Zhejiang Topo Co. Ltd., China). Fresh weight was measured after removing surface dirt and blotting excess water from the roots. Seedlings were then placed in an envelope, killed at 180°C for 30 min and dried at a constant temperature of 80°C for 12 h to determine the dry weight.

### 4.6 Chlorophyll Content Measurement

Chlorophyll (Chl) extraction was performed by immersing 0.1 g of fresh leaves in 10 mL of 80% acetone (v/v) until the leaves turned white. The chlorophyll content was quantified using a UV‐1800 spectrophotometer (Shimadzu, Japan) and calculated according to the protocol described by Moustakas et al. (2022).

### 4.7 NO Content Measurement

Tomato leaves (0.3 g) were ground with liquid nitrogen and homogenised with 1.5–2 mL of extraction solution (50 mM glacial acetic acid buffer containing 4% zinc diacetate). The mixture was centrifuged at 10000 ***g*** for 15 min at 4°C. The supernatant was treated with 0.1 g of activated charcoal, vortexed and filtered through filter paper to obtain the reaction solution.

For the assay, 1 mL of the reaction solution was mixed with 1 mL of Griess reagent and incubated at room temperature for 30 min. Absorbance was measured at 540 nm using a spectrophotometer (Shimadzu, Japan). A control was prepared by mixing the extraction solution with Griess reagent and sterilised distilled water was used for baseline adjustment.

### 4.8 NO Fluorescent

The DAF‐FM DA probe provided in the kit was diluted at a ratio of 1:1000 to achieve a final concentration of 5 μmol L^−1^. The diluted solution was prepared in a brown centrifuge tube and protected from light to prevent degradation. The roots and cross‐sections of the sample were submerged in the diluted DAF‐FM DA solution. To ensure full contact between the probe and the root system, the tube was gently inverted every 3–5 min during incubation. After incubation, the roots were washed with PBS (pH 7.4) to remove excess probe. The samples were observed and photographed using an LSM800 confocal laser scanning microscopy (CLSM, Carl Zeiss Microscopy, Hamburg Germany) with excitation at 495 nm and emission at 515 nm. The green fluorescence signal intensities in the captured images were quantified using IMAGEJ software (National Institutes of Health, Bethesda, MD, USA).

### 4.9 Measurement of SNO Content and GSNOR Activity

SNO content was measured using the method described by Gong et al. (2015), with modifications. Samples were lysed in liquid nitrogen by adding 3 mL of extraction buffer containing 1 mM PMSF and incubated on ice for 20 min. The supernatant was then collected by centrifugation at 10000 ***g*** for 15 min at 4°C. For the control, 700 μL of the supernatant was mixed with an equal volume of liquid A (0.5 mol L^−1^ HCl with 1% sulfanilamide). Separately, 1.5 mL of the supernatant was mixed with an equal volume of liquid B (liquid A with 0.2% HgCl_2_). The remaining extract was reserved for protein concentration determination. Next, 1.4 mL of liquid C (containing 0.1% N‐(1‐naphthyl) ethylenediamine) was added to the tube containing liquid A, and 3 mL of liquid C was added to the tube containing liquid B. The mixtures were incubated at 37°C for 10 min. Absorbance at 540 nm was measured using a 1 mL cuvette (three replicates). SNO content was determined as the difference in absorbance between the reaction solutions of liquid B and liquid A. GSNO concentration was derived from the absorbance of liquid B. To quantify SNO, a standard curve was generated by measuring the absorbance at 540 nm using different concentrations of GSNO instead of protein extract. SNO content was calculated using the standard curve equation.

GSNOR activity was measured as follows: tomato samples were powdered with liquid nitrogen and homogenised in 1.4 mL of 50 mM HEPES buffer (pH 8.0). The homogenate was centrifuged at 12000 ***g*** for 15 min at 4°C, and the supernatant was collected. A reaction mixture was prepared by adding 30 μL of protein sample to 180 μL of 0.1 M phosphate buffer containing 10 μL of 6 mM NADPH and 10 μL of 6 mM GSNO. Absorbance at 340 nm was measured immediately after adding NADPH for 1 min.

### 4.10 In Vivo Protein *S*‐Nitrosylation Assay

The in vivo *S*‐nitrosylation assay was conducted using the biotin transformation method, with minor modifications as described by Feng, Wang, et al. (2013). Tomato seedlings were homogenised in HEN2 buffer (250 mM HEPES, pH 7.7, 1 mM EDTA, 0.1 mM neocuproine, 1% protease inhibitor cocktail) and incubated on ice for 30 min. The extract was centrifuged twice at 13000 ***g*** for 15 min at 4°C, and the supernatant was collected for protein concentration determination using the Protein Assay Kit (Thermo Scientific). For the assay, 0.5 mg of protein sample was mixed with blocking buffer (250 mM HEPES, pH 7.7, 1 mM EDTA, 0.1 mM neocuproine, 2.5% SDS, 25 mM MMTS) and incubated at 50°C for 30 min in a shaker to block free cysteine residues. Excess MMTS was removed with 1 mL acetone, and the protein was precipitated by centrifugation at 13000 *g* for 10 min after incubation at −20°C. The protein was resuspended in 800 μL HENS buffer (250 mM HEPES, pH 7.7, 1 mM EDTA, 0.1 mM neocuproine, 1% SDS) and reacted with 1 M sodium ascorbate and 4 mM biotin‐HPDP in the dark at room temperature. After protein precipitation, the sample was suspended in 400 μL HENS buffer and incubated overnight at 4°C to ensure complete resuspension. Next, 800 μL neutralisation buffer and 30 μL streptavidin agarose (Thermo Fisher Scientific, USA, Cat #: 20349) were added to facilitate protein binding. All steps were carried out in the dark. The beads were washed 5–6 times with washing buffer (25 mM HEPES, pH 7.7, 60 mM NaCl, 1 mM EDTA, 0.5% Triton X‐100) to remove non‐specific interactions. Modified proteins were eluted in 20 μL elution buffer (250 mM HEPES, pH 7.7, 1 mM EDTA, 0.1 mM neocuproine, 1% cocktail, 1% β‐mercaptoethanol) and denatured by boiling with 6 × SDS‐PAGE loading buffer for 5 min. Finally, the samples were separated on 12% SDS‐PAGE gels for further analysis.

### 4.11 Recombinant Protein Expression

The coding sequences of MEK1, MEK1^C172S^ and MEK1^C172W^ were cloned into the pET‐30a vector and transformed into Transetta (DE3) *Escherichia coli* . The bacteria were cultured in LB medium supplemented with 100 μg mL^−1^ kanamycin and chloramphenicol until the OD600 reached 0.6–0.8. Protein expression was induced by adding 0.1 mM IPTG, and the cultures were incubated at 16°C for 16 h. The cells were harvested by centrifugation and resuspended in low‐salt buffer supplemented with PMSF, Triton‐X 100 and lysozyme. After incubation at room temperature, the cells were lysed by sonication and centrifuged to remove debris. The supernatant was filtered and mixed with Ni‐NTA resin, and the mixture was incubated at 4°C for 1–2 h to allow protein binding. The bound proteins were eluted using a stepwise gradient of imidazole concentrations, concentrated by ultrafiltration and stored at −80°C for further use.

### 4.12 In Vitro Protein *S*‐Nitrosylation Assay

The in vitro *S*‐nitrosylation assay was performed with minor adjustments (Feng, Wang, et al. 2013). Briefly, 30 μg of His‐tagged MEK1, MEK1^C172W^ and MEK1^C172S^ recombinant proteins were incubated with 200 μM GSNO in the dark for 1 h. The proteins were then acetone‐precipitated and resuspended in 300 μL blocking buffer I (250 mM HEPES, pH 7.7, 4 mM EDTA, 0.1 mM neocuproine, 2.5% [v/v] SDS and 0.1% [v/v] S‐methylmethane thiosulfonate). After incubation at 50°C for 30 min, the proteins were precipitated and dissolved in 80 μL HENS buffer (250 mM HEPES, pH 7.7, 4 mM EDTA, 0.1 mM neocuproine, 1% [v/v] SDS) with the addition of 10 μL 500 mM sodium ascorbate (Asc) and 10 μL of 4 mM biotin‐HPDP. The reaction proceeded at room temperature for 1 h. The samples were separated by SDS–PAGE without boiling and analysed by immunoblotting using anti‐His antibody (Cat#E12‐004‐3, EnoGene, 1:2000) for input and anti‐biotin antibody (Cat#7075, Cell Signalling Technology, 1:2000) to detect *S*‐nitrosylated MEK1, MEK1^C172W^ and MEK1^C172S^.

Potential *S*‐nitrosylation sites in the MS‐identified MEK1 protein were predicted using the GPS‐SNO (Group‐Based Prediction System, http://sno.biocuckoo.org).

### 4.13 Na^+^ and K^+^ Content Assay

To estimate Na^+^ and K^+^ content, tomato plants were treated with 150 mM NaCl for 7 days. On day 7, seedlings were harvested, dried at 80°C for 3 days and 0.1 g dry sample was digested in 2 mL 10 mM HNO_3_. The volume was adjusted to 10 mL using distilled water. Na^+^ and K^+^ concentrations were measured using an Optima 2100DV inductively coupled plasma optical emission spectrometer (ICP‐OES, PerkinElmer, USA). The experiment was repeated three times for reliability.

### 4.14 Salt Tolerance Index Measurement

At the end of the treatment period, plants from both the control and salt‐stress groups were harvested simultaneously. Entire plants were placed in an envelope and dried in an oven at 80°C for 48 h until a constant weight was achieved. The total dry weight was then recorded. The tolerance index (TI) for each genotype or treatment was calculated using the following formula:

$$\begin{array}{c} \mathrm{TI}\;\left(\%\right)=\left(\text{Total}\;\mathrm{dry}\;\text{weight under salt stress}/\right.\\ \left.\text{Total}\;\mathrm{dry}\;\text{weight under control conditions}\right)\times 100\% \end{array}$$

### 4.15 Co‐Immunoprecipitation (Co‐IP) Assay

MEK1 and GSNOR were cloned into GFP‐ and HA‐tagged vectors, respectively, and the plasmids were transformed into *Agrobacterium tumefaciens* . After centrifugation, the cells were resuspended in an infection solution (10 mM MgCl_2_, 10 mM MES and 200 μM acetosyringone) and infiltrated into tobacco leaves at a 1:1 (v/v) ratio with an OD600 of 0.8–1.0. Leaves were collected 48 h post‐infiltration, and proteins were extracted for Western blotting to confirm expression. For co‐immunoprecipitation (Co‐IP) analysis, samples were washed with IP lysis/washing buffer, centrifuged and eluted for SDS‐PAGE (Ren et al. 2013). The primers used for cloning are listed in Table S2.

### 4.16 Bimolecular Fluorescence Complementation Assay (BiFC)

The *MEK1* gene was cloned into the pCAMBIA1300‐35 s‐C‐YFPC vector, and the *GSNOR* gene was cloned into the pCAMBIA1300‐35 s‐N‐YFPN vector. *A. tumefaciens* GV3101 cells harbouring these plasmids were infiltrated into 4‐week‐old *N*. *benthamiana* leaves. YFP signals were detected using laser scanning confocal microscopy 4 days post‐infiltration.

For imaging, 0.5 cm tobacco leaf segments were collected, placed on glass slides and stained with DAPI staining solution (Solabio, C0065; 10 μg mL^−1^) for 5–10 min. The staining solution was removed, and the samples were washed at least three times with PBS (pH 7.4) to eliminate unabsorbed dye. Coverslips were applied, and samples were observed and imaged using an S‐3400 N SEM (Hitachi, Japan). The maximum excitation and emission wavelengths were set at 340 nm and 488 nm, respectively. The 488 nm laser line was also used to excite chlorophyll in chloroplasts. The primers used are shown in Table S2.

### 4.17 Yeast Two‐Hybrid (Y2H) Assay

The full‐length MEK1 and GSNOR sequences were cloned into pGBKT7 and pGADT7 vectors, respectively, for transactivation assays and protein interaction studies. The recombinant plasmids, pGBKT7‐MEK1 and pGADT7‐GSNOR, were transformed into yeast strains using the LiAc‐PEG method. Cotransformants were selected on SD (−Leu/−Trp) medium, and protein interactions were assessed by growth on SD (−Leu/−Trp/−His/−Ade) medium. Positive and negative controls included pGADT7‐T with pGBKT7‐53 and pGBKT7‐Lam, respectively (Ni et al. 2021). The primers used are listed in Table S2. To investigate the effect of NO on interaction strength, 10 μM NO donors (GSNO and SNP) were added to medium alone. Yeast growth capacity was found to be positively correlated with the strength of the interaction.

### 4.18 Luciferase (LUC) Assay

Full‐length cDNAs of MEK1 and GSNOR were cloned into pCAMBIA1300‐nLUC and pCAMBIA1300‐cLUC vectors, respectively. *A. tumefaciens* was transformed with these constructs to prepare the infiltration solution, following the same protocol as for subcellular localization. Equal volumes of the target vectors (nLUC and cLUC) or empty vector control were mixed and incubated in the dark at room temperature for 3 h. The solution was infiltrated into the abaxial surface of *N*. *benthamiana* leaves using a 1 mL syringe. After 4 days of incubation in the greenhouse, leaf sections were cut, sprayed with 1 × D‐Luciferin (Solebol), and luminescence was visualised using a CCD image analyser (ANDOR, Live Imaging System). The primers used are shown in Table S2.

### 4.19 Statistical Analysis

Data was analysed using Excel 2016 and SPSS 24.0 (SPPS Inc. Chicago, IL, USA). Results are expressed as means ± standard deviation (SD) of three independent experiments, each performed in triplicate. Statistical differences between treatments were assessed using Duncan's multiple range test at a significance level of *p* < 0.05 levels.

## Author Contributions

W.L., C.W. and H.F. conceived and designed the research; H.F., H.C., D.H. and X.W. performed the experiments. X.C., Z.L., X.P. and H.F. analysed the data; X.H. and S.W. methodology, investigation, formal analysis. H.F., D.Z. and C.M. wrote the paper. W.L. review and editing, formal analysis, supervision, funding acquisition, conceptualization.

## Conflicts of Interest

The authors declare no conflicts of interest.

## Supporting information


**Appendix S1:** pbi70585‐sup‐0001‐AppendixS1.zip.

**Figure S1:** pbi70585‐sup‐0002‐FiguresS1‐S15.docx.

**Table S1:** pbi70585‐sup‐0003‐TablesS1‐S3.docx.

## Data Availability

The data that supports the findings of this study are available in Appendix S1, Figures S1–S15 and Tables S1–S3 of this article.
