# Supplementary material for: Hydrogen Gas Enhances Salinity Tolerance in Tomato Seedlings by Regulating the S‐Nitrosylation of MEK1
Source: Plant Biotechnol J. 2026 Feb 18;24(6):3656–78. doi: 10.1111/pbi.70585 (PMC13205882; doi:10.1111/pbi.70585)
Supplement: Supplementary file 2 — Figure S1: pbi70585‐sup‐0002‐FiguresS1‐S15.docx. [file PBI-24-3656-s002.docx]

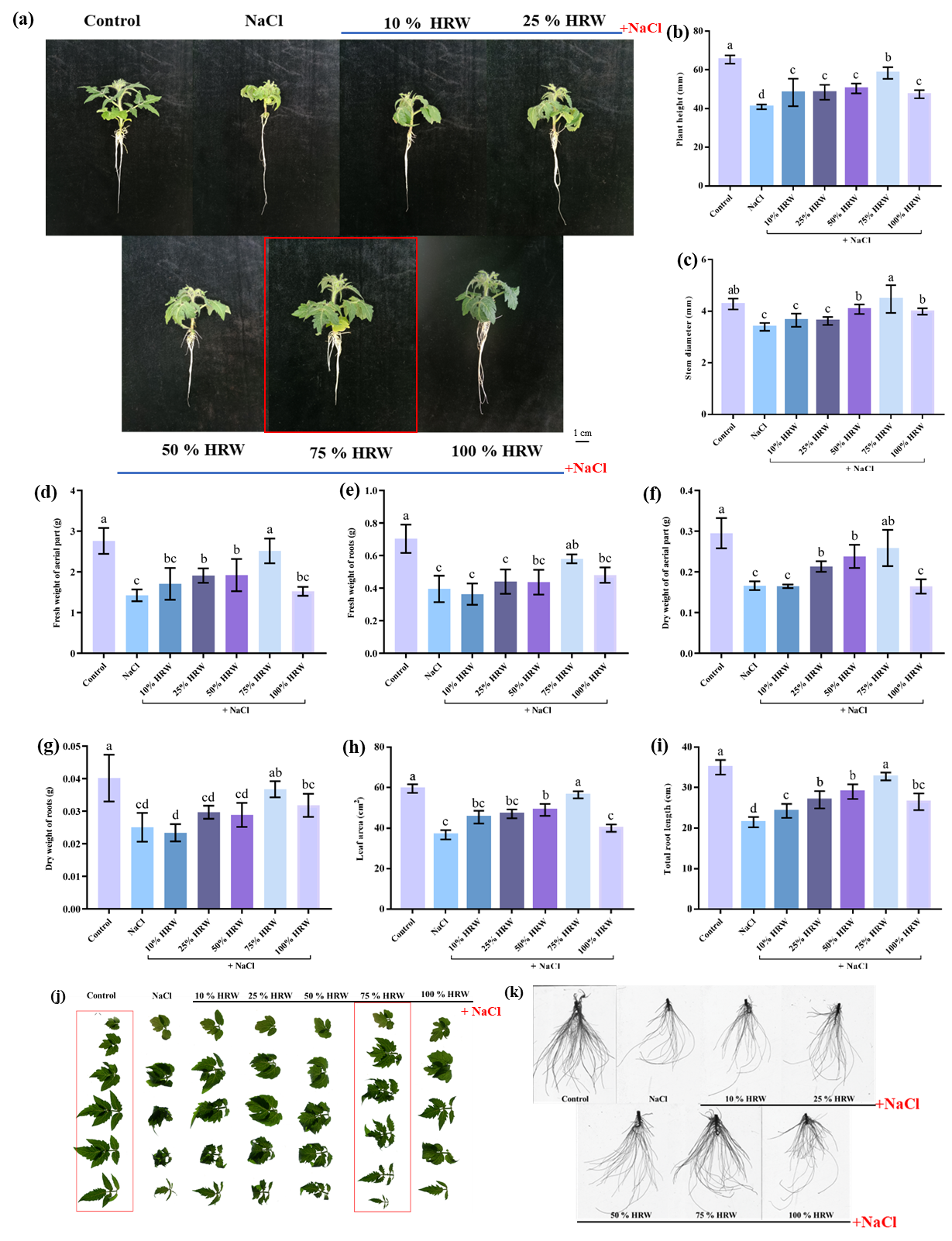


Figure S1 H_2_ alleviated salt stress in an H_2_-dependent manner. Photographs were taken after 7 days of the treatments indicated. Phenotypic analysis of tomato seedlings (a), plant height (b), stem diameter (c), fresh weight of aerial part (d), fresh weight of roots (e), dry weight of aerial part (f) and dry weight of roots (g), leaf area (h, j) and total root length (I, k) under different concentration of HRW. Data are expressed as mean ± SD (n = 3), and experiments were performed in triplicate. Different letters indicate significant differences (*P* < 0.05), as determined by Duncan’s multiple range test.


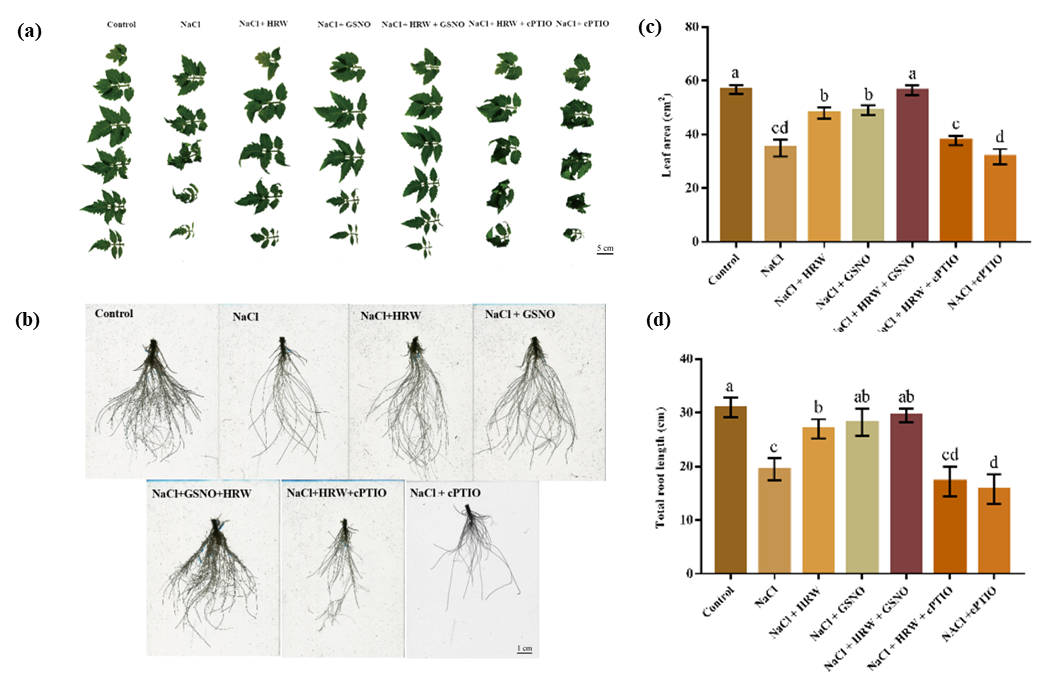


Figure S2. Effects of NO scavenger cPTIO on the leaf area (a, c) and total root length (b, d) of tomato seedlings under NaCl stress. Measurements were taken 7 d post-treatment. Data are expressed as mean ± SD (n = 3), and experiments were performed in triplicate. Different letters indicate significant differences (*P* < 0.05), as determined by Duncan’s multiple range test.


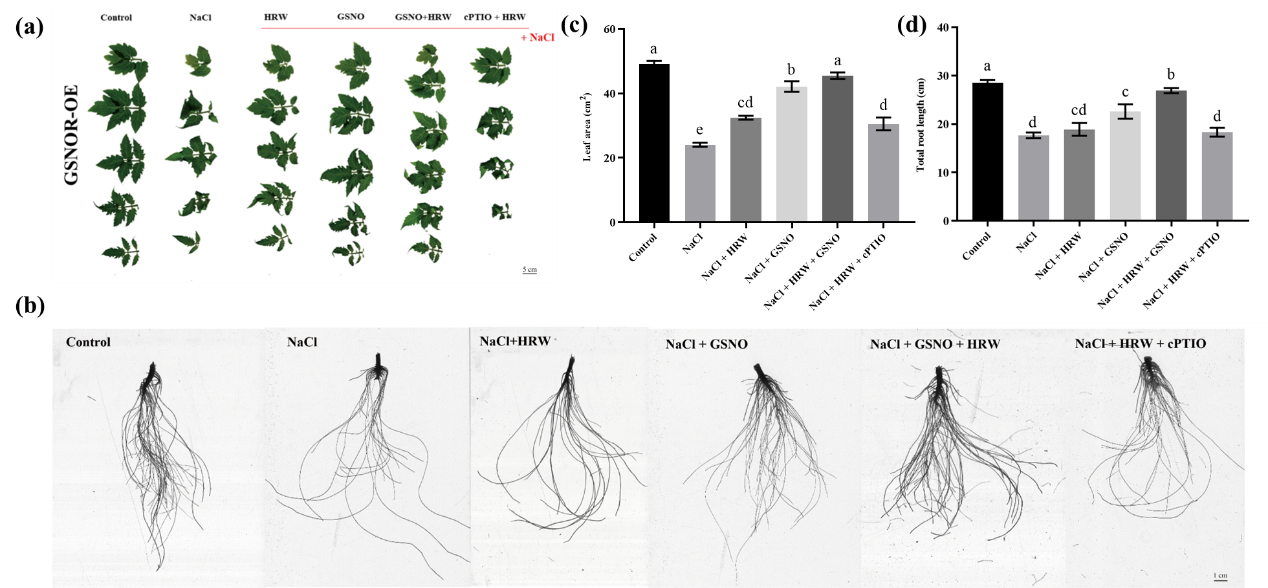


Figure S3 Effects of NO scavenger cPTIO on the leaf area (a, c) and total root length (b, d) of *SlGSNOR* overexpression (*SlGSNOR*-OE) tomato seedlings under NaCl stress. Measurements were taken 7 d post-treatment. Data are expressed as mean ± SD (n = 3), and experiments were performed in triplicate. Different letters indicate significant differences (*P* < 0.05), as determined by Duncan’s multiple range test.


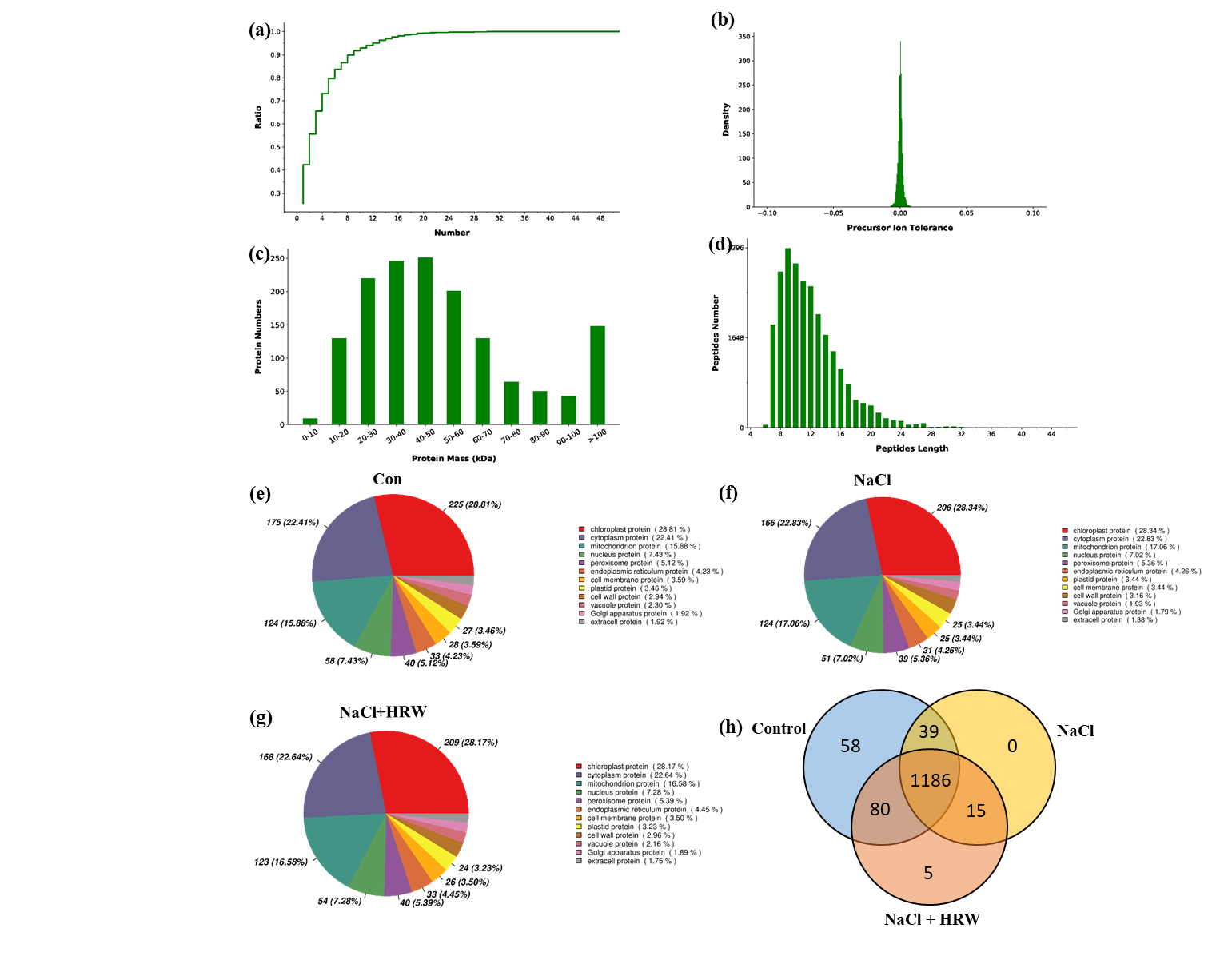


Figure S4 Identification and analysis of *S*-nitrosylated proteins in tomato seedling. Quality analysis of mass spectrometry data (a-d), intracellular localization of *S*-nitrosylated proteins (e-g) and Venn diagram (h) under different treatments.


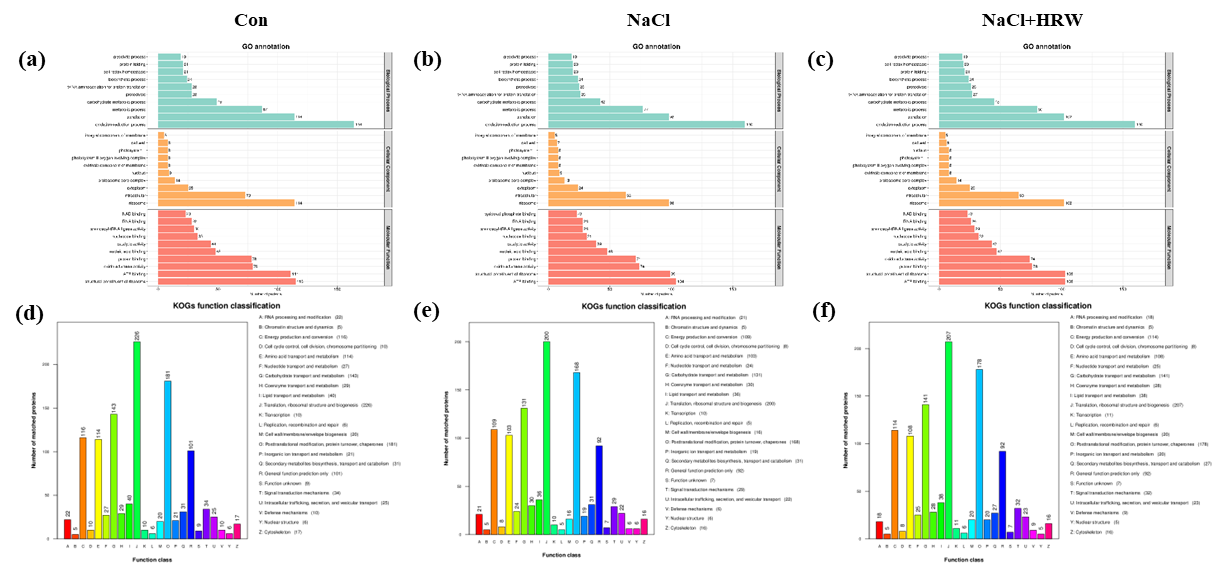


Figure S5 Functional comparison of *S*-nitrosylated proteins in different treatments based on GO (a-c) and GOC (d-f) annotation


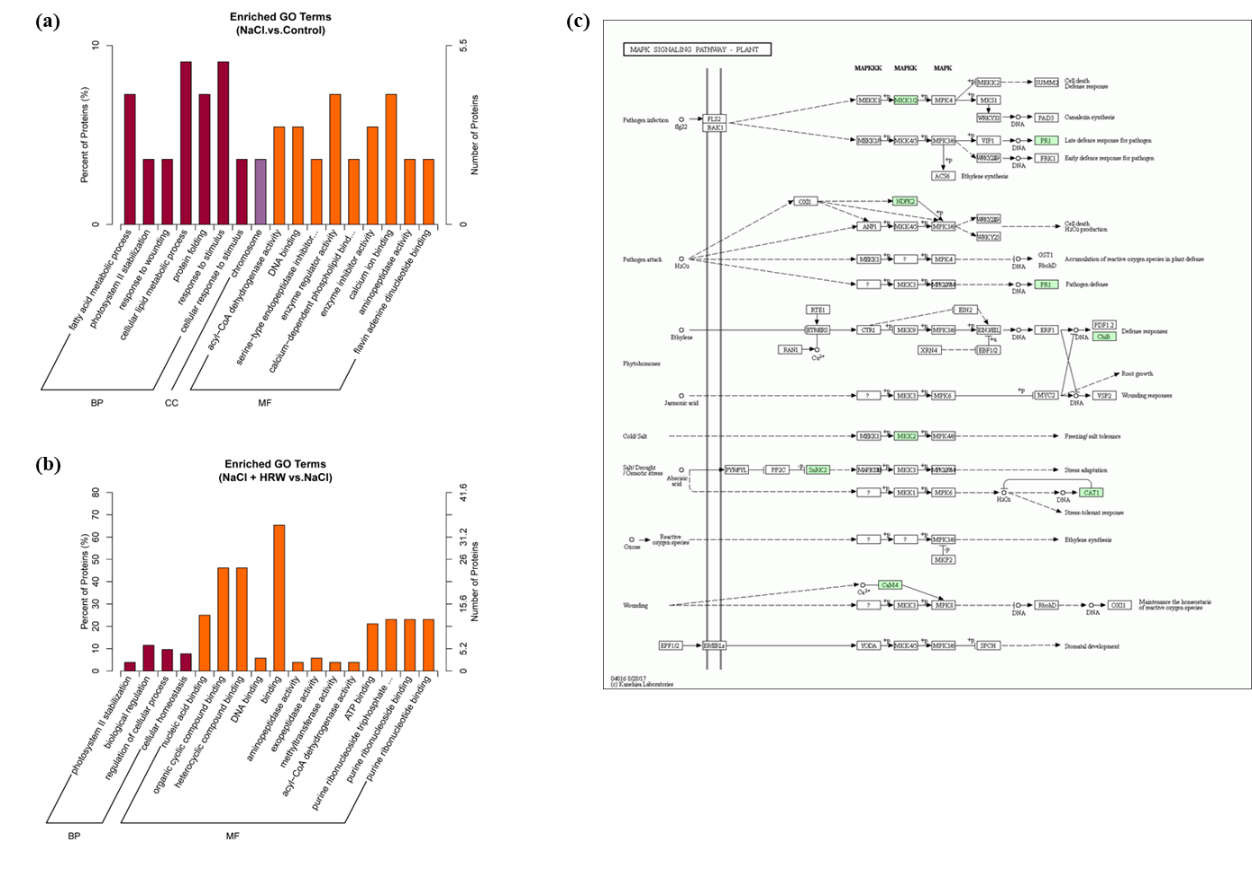


Figure S6 HRW alleviates salt stress-triggered proteome *S*-nitrosylation. GO annotation of all upregulated *S*-nitrosylated proteins in “NaCl vs. Control” and all downregulated *S*-nitrosylated proteins in “NaCl + HRW vs. NaCl” (a-b). *S*-nitrosylated proteins in the MAPK related pathways (c). *S*-nitrosylated enzymes or proteins are highlighted in green (see Table 1 with accession numbers and detailed annotations of these proteins).


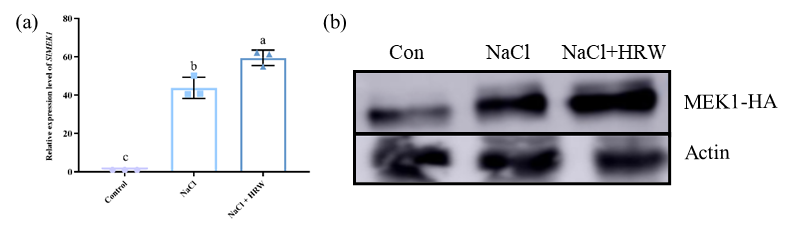


Figure S7 Expression levels of the MEK1 gene (a) and protein (b) under different treatments. Data are expressed as mean ± SD (n = 3), and experiments were performed in triplicate. Different letters indicate significant differences (*P* < 0.05), as determined by Duncan’s multiple range test.


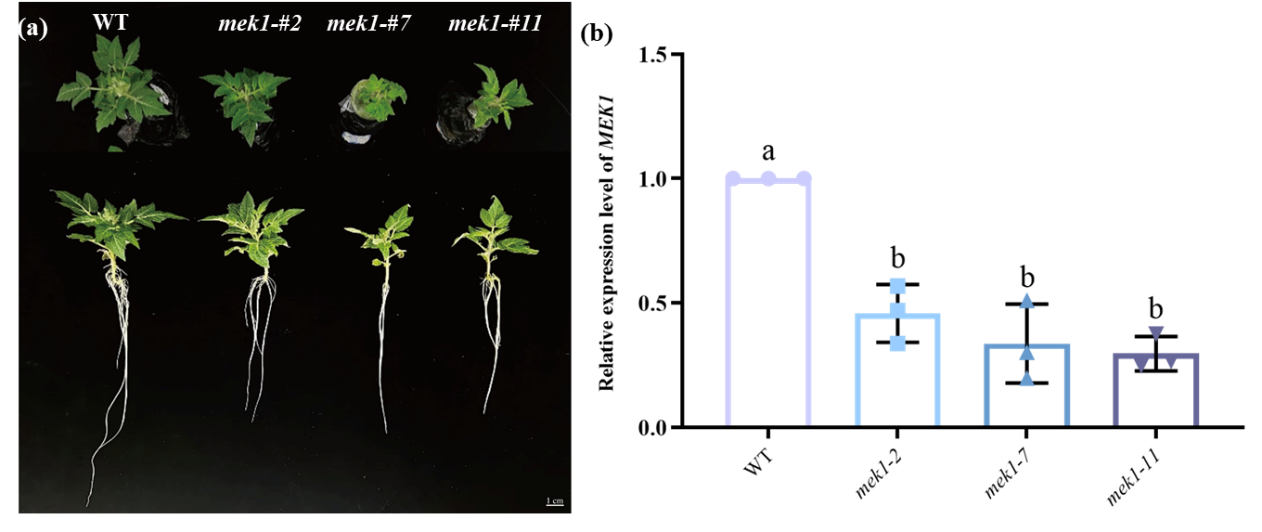


Figure S8 Photographs of *mek1* lines grown (a) and quantitative RT-PCR analysis of *SlMEK1* in WT and *mek1* transgenic plants (b). Data are expressed as mean ± SD (n = 3), and experiments were performed in triplicate. Different letters indicate significant differences (*P* < 0.05), as determined by Duncan’s multiple range test.


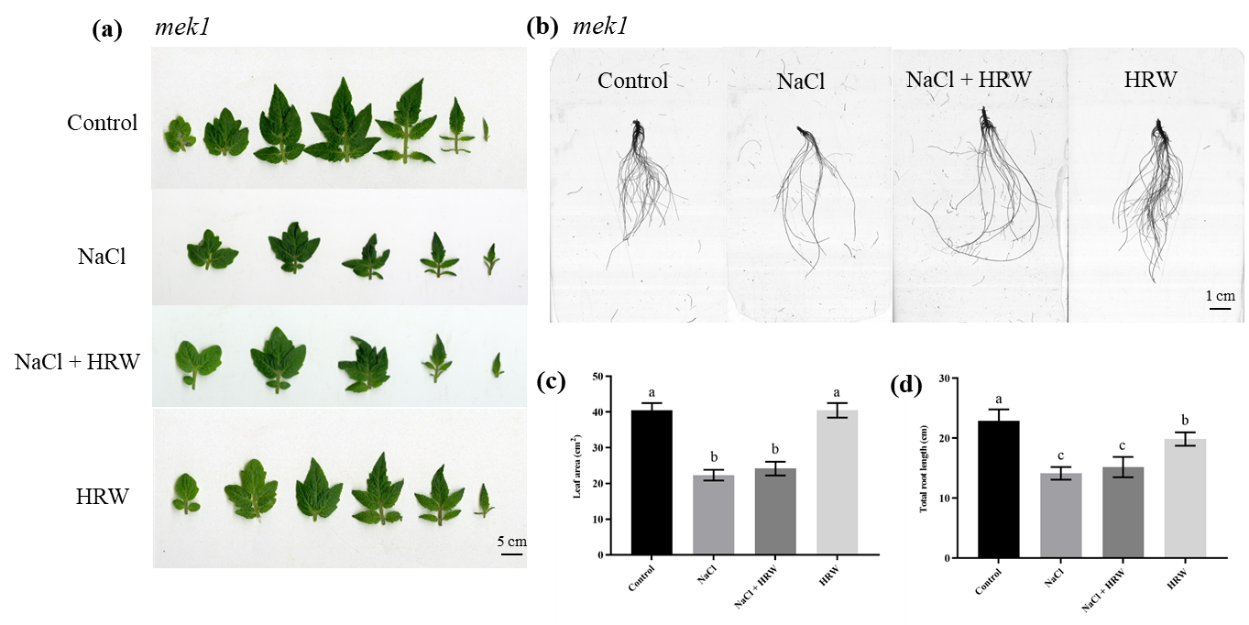


Figure S9 Effect of *SlMEK1* knockout on the leaf area and total root length of tomato seedlings under NaCl stress. Photographs of leaf area (a), photographs of root (b), leaf area (c), total root length (d). Measurements were taken after 7 d post-treatments. Data are expressed as mean ± SD (n = 3), and experiments were performed in triplicate. Different letters indicate significant differences (*P* < 0.05), as determined by Duncan’s multiple range test.


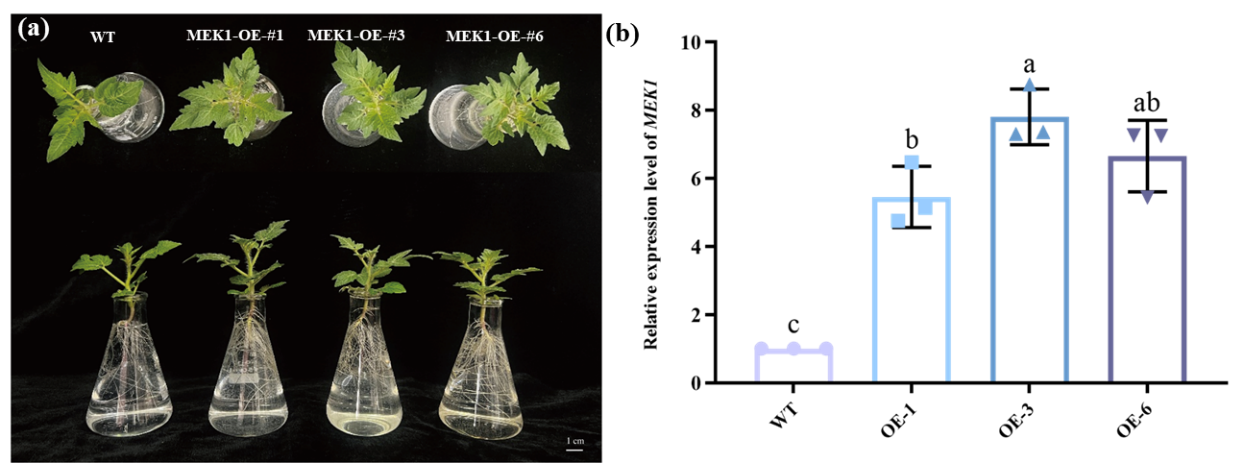


Figure S10 Photographs of *SlMEK1* overexpression (OE) lines grown (a) and quantitative RT-PCR analysis of *SlMEK1* in WT and MEK1-OE transgenic plants (b).


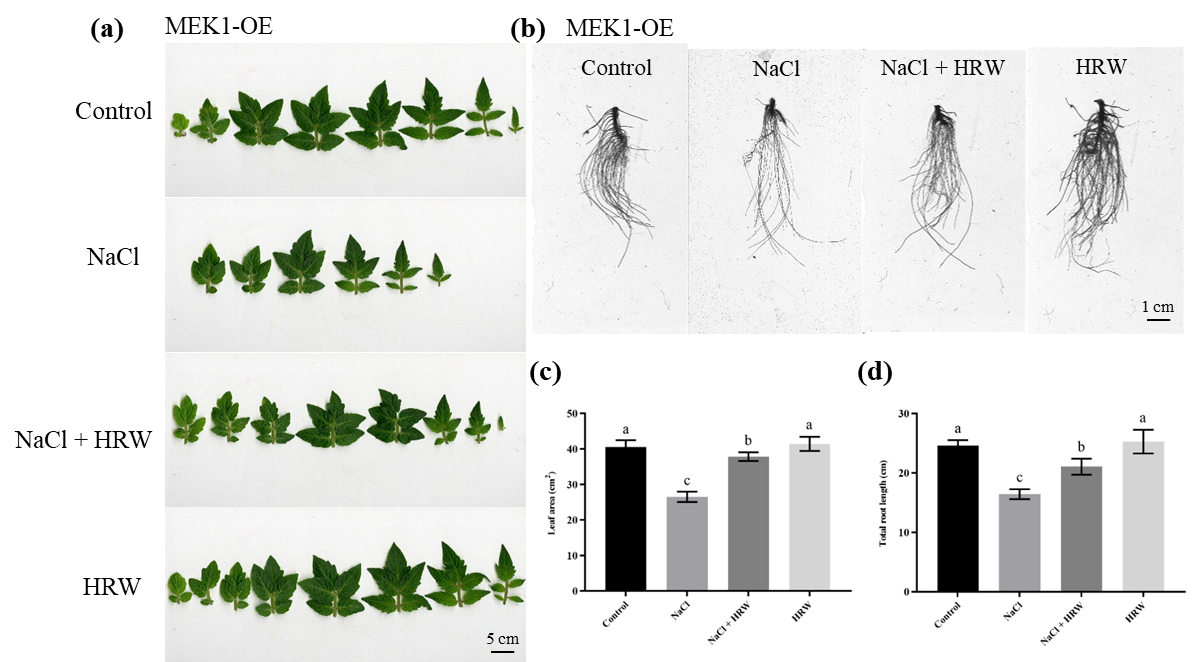


Figure S11 Effect of *SlMEK1* overexpression on the leaf area and total root length of tomato seedlings under NaCl stress. Photographs of leaf area (a), photographs of root (b), leaf area (c), total root length (d). Measurements were taken 7 d post-treatment. Data are expressed as mean ± SD (n = 3), and experiments were performed in triplicate. Different letters indicate significant differences (*P* < 0.05), as determined by Duncan’s multiple range test.


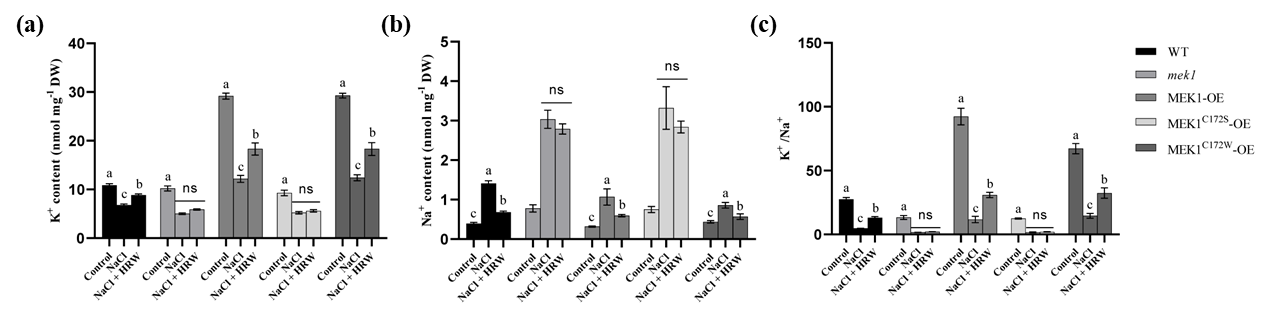


Figure S12 Na^+^ and K^+^ contents in WT and *SlMEK1* genetic materials under 150 mM NaCl. K^+^ content (a), Na^+^ content (b), K^+^/ Na^+^ ratio (c), in shoot of the plant. Measurements were taken 7 d post-treatment. Data are expressed as mean ± SD (n = 3), and experiments were performed in triplicate. Different letters within the same line indicate significant differences (*P* < 0.05), as determined by Duncan’s multiple range test.


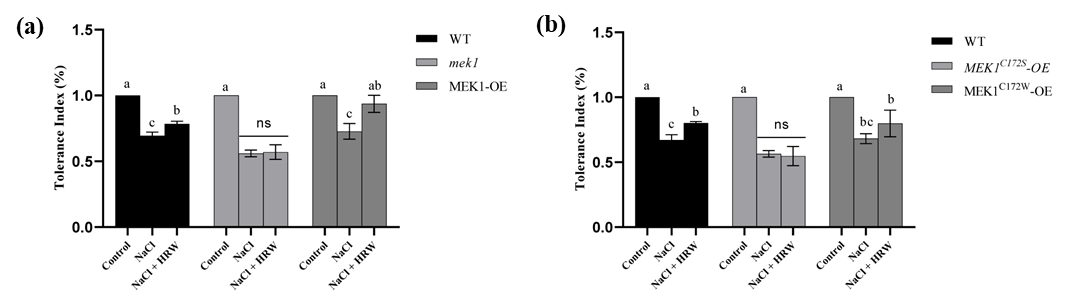


Figure S13 Tolerance index in WT and *SlMEK1* genetic materials under 150 mM NaCl. Tolerance index in WT, *mek1*, and *MEK1*-OE tomato seedlings (a), Tolerance index in WT, *MEK1^C172S^*-OE, and *MEK1 ^C172W^* -OE tomato seedlings (b). Measurements were taken 7 d post-treatment. Data are expressed as mean ± SD (n = 3), and experiments were performed in triplicate. Different letters within the same line indicate significant differences (*P* < 0.05), as determined by Duncan’s multiple range test.


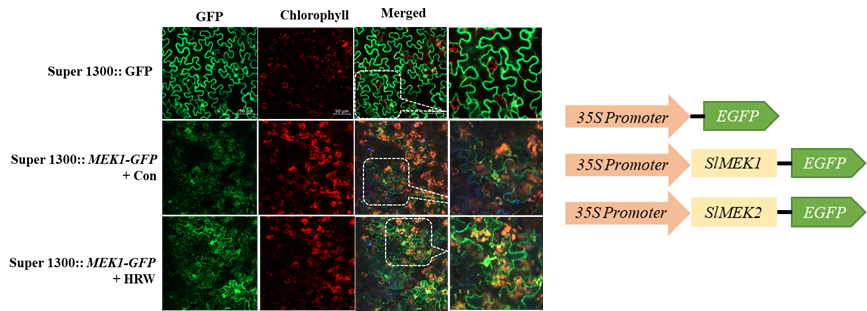


Figure S14 Effects of HRW on the subcellular localization of MEK1 in Tobacco. The indicated constructs were co-expressed in tobacco epidermal leaves. Scale bar, 50 μm.


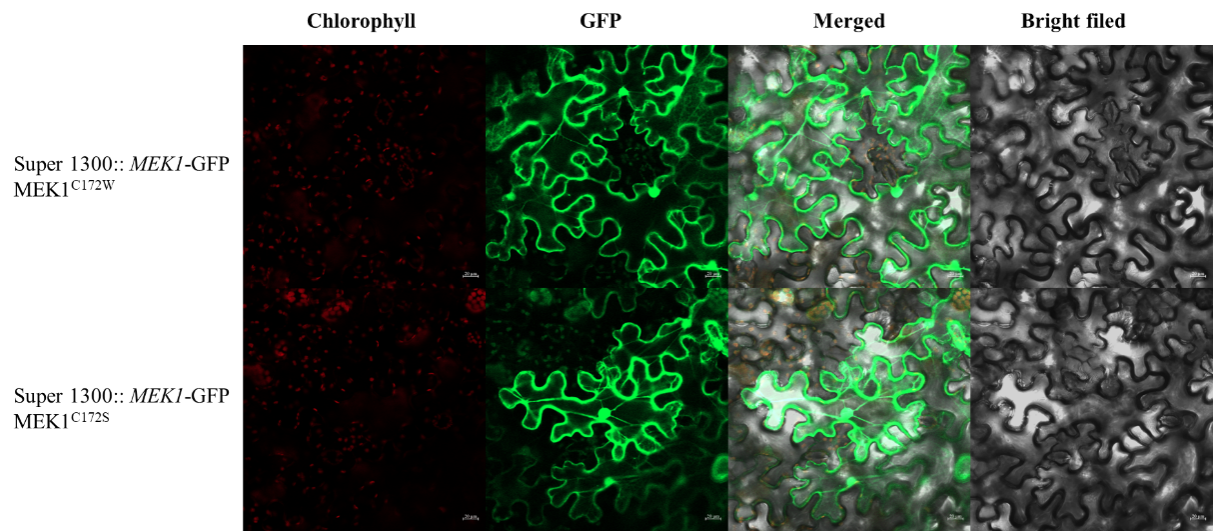


Figure S15 Effect of mutation at cys172 on MEK1 subcellular localization. The indicated constructs were co-expressed in tobacco epidermal leaves. Scale bar, 20 μm.
