# Supplementary material for: Hydrogen Gas Enhances Salinity Tolerance in Tomato Seedlings by Regulating the S‐Nitrosylation of MEK1
Source: Plant Biotechnol J. 2026 Feb 18;24(6):3656–78. doi: 10.1111/pbi.70585 (PMC13205882; doi:10.1111/pbi.70585)
Supplement: Supplementary file 3 — Table S1: pbi70585‐sup‐0003‐TablesS1‐S3.docx. [file PBI-24-3656-s003.docx]

Supplemental Table 1 *S*-nitrosylated proteins under NaCl + HRW vs NaCl + GSNO

| A0A3Q7J601 | A0A3Q7IIS5 | A0A3Q7F7L6 | A0A3Q7F9M7, | A0A3Q7ENP1 | A0A3Q7JE01 | A0A3Q7G087 |
| --- | --- | --- | --- | --- | --- | --- |
| A0A3Q7HCH3 | A0A3Q7GNI9 | A0A3Q7J0I3 | A0A3Q7HCH3 | A0A3Q7GNI9 | A0A3Q7HYJ1 | A0A3Q7EUN5 |
| A0A3Q7EQ90 | A0A3Q7J2K1 | K4DH15 | A0A3Q7IFK3 | Q5QJB4 | A5JV19 | A0A3Q7GLY3 |
| A0A3Q7I752 | A0A3Q7HFP1 | A0A3Q7HWN1 | A0A3Q7J2Z9 | A0A3Q7J3Q6 | A0A3Q7J5H8 | B5M9E5 |
| A0A3Q7HNG4 | A0A3Q7I738 | A0A3Q7GMW1 | A0A3Q7FV93 | A0A3Q7GBR4 | A0A3Q7GP23 | A0A3Q7ENE9 |
| A0A3Q7IMD9 | A0A3Q7FWL3 | A0A3Q7IRS4 | A0A3Q7JW47 | A0A3Q7GE81 | A0A3Q7F688 | A0A3Q7IQJ3 |
| A0A3Q7IP94 | Q8RVV4 | A0A3Q7GBI5 | A0A3Q7JD76 | A0A3Q7IC89 | A0A3Q7FFY2 | A0A3Q7GSM2 |
| A0A3Q7FCR4 | A0A3Q7FN98 | A0A3Q7HH10 | A0A3Q7EHP2 | A0A3Q7GPV9 | K4D4H5 | A0A3Q7I6B5 |
| P27065 | A0A3Q7E9J6 | A0A3Q7FCZ7 | A0A3Q7GJV0 | A0A3Q7HEC9 | A0A3Q7IZ03 | O65004 |
| A0A3Q7FAJ4 | A0A3Q7G7L7 | A0A3Q7I9X4 | A0A3Q7F898 | A0RZC9 | A0A3Q7IBR0 | A0A3Q7GCI2 |
| A0A3Q7H3P6 | A0A3Q7GK31 | P93212 | A0A3Q7HWB4 | A0A3Q7ETK4 | A0A3Q7GRY3 | A0A3Q7H4X3 |
| A0A3Q7HHL0 | A0A3Q7I0P8 | A0A3Q7IT31 | A0A3Q7JCN9 | A0A3Q7IHG3 | A0A3Q7GYZ2 | A0A3Q7G730 |
| A0A3Q7JMX3 | Q93X45 | A0A3Q7ILD3 | A0A3Q7I8G1 | A0A3Q7JPK6 | A0A3Q7FUV3 | A0A3Q7G7H8 |
| Q672Q9 | A0A3Q7JSG3 | A0A3Q7IHL0 | A0A3Q7HEZ3 | A0A3Q7HNB6 | D1MAF2 | A0A3Q7I8W8 |
| A0A3Q7I7U0 | A0A3Q7FU69 | A0A3Q7I009 | O65917 | A0A3Q7I8A0 | A0A3Q7GN27 | A0A3Q7H1L7 |
| A0A3Q7ESW4 | A0A3Q7H112 | A0A3Q7EFC3 | O81536 | A0A3Q7IL38 | A0A3Q7GM93 | A0A3Q7FCM3 |
| A0A3Q7FF67 | A0A3Q7J4V1 | Q645M9 | A0A3Q7EN49 | A0A3Q7HGI8 | A0A3Q7I245 | A0A3Q7J033 |
| A0A3Q7HGD7 | A0A3Q7HW79 | A0A3Q7HCM3 | A0A3Q7FRB5 | A0A3Q7IKY6 | A0A3Q7I9A2 | A0A3Q7HE03 |
| A0A3Q7G5E6 | A0A3Q7FBU5 | A0A3Q7J1M2 | P43282 | A0A3Q7I1J2 | A0A3Q7HR13 | A0A3Q7HGC8 |
| A0A3Q7EQ44 | A0A3Q7F8V6 | A0A3Q7FBR1 | A0A3Q7GM08 | Q5NE18 | A0A3Q7GVZ8 | A0A3Q7FMZ4 |
| A0A3Q7HWL8 | A0A3Q7H2U0 | Q43775 | A0A3Q7JF63 | A0A3Q7IN91 | A0A3Q7HQA3 | A0A3Q7GX01 |
| A0A3Q7EUX7 | Q2MI93 | A0A3Q7GB48 | A0A3Q7FWE8 | A0A3Q7JA27 | Q42884 | A0A3Q7EZ16 |
| A0A3Q7HQH3 | A0A3Q7IPT1 | A0A3Q7E7B4 | A0A3Q7GSZ7 | A0A3Q7I7R0 | A0A3Q7F5F8 | A0A3Q7GD89 |
| A0A3Q7ICW3 | A0A3Q7HTC1 | A0A3Q7JI81 | A0A3Q7J0Z8 | A0A3Q7EKQ6 | A0A3Q7HCG1 | A0A3Q7FYW1 |
| A0A3Q7HIP9 | A0A3Q7JF55 | A0A3Q7I2I7 | O48616 | A0A3Q7HC76 | A0A3Q7IFC2 | A0A3Q7FCE4 |
| A0A3Q7E8A8 | A0A3Q7GKS5 | A0A3Q7F7G5 | A0A3Q7JA97 | A0A3Q7G077 | A0A3Q7GG74 | A0A3Q7HE62 |
| A0A3Q7GQA6 | A0A3Q7FN07 | A0A3Q7FY87 | A0A3Q7GP62 | A0A3Q7H1L0 | A0A3Q7JAS5 | A0A3Q7J365 |
| A0A3Q7HG98 | L7Q568 | A0A3Q7HG29 | A0A3Q7EHA9 | A0A3Q7EF20 | A0A3Q7IZQ5 | Q6R8F6 |
| A0A3Q7IHS3 | A0A3Q7HIJ0 | A0A3Q7F8X1 | A0A3Q7E824 | K4BF05 | A0A3Q7EU46 | A0A3Q7GTM3 |
| A0A3Q7GJR0 | A0A3Q7GH43 | A0A3Q7HH02 | A0A3Q7G465 | A0A3Q7IPS6 | A0A3Q7FD04 | A0A3Q7EQP9 |
| A0A3Q7IQR1 | Q6SKP4 | A0A3Q7HWG9 | A0A3Q7EM55 | A0A3Q7I4P2 | A0A3Q7HBK4 | A0A3Q7FSD7 |
| A0A3Q7F352 | A0A3Q7EPZ7 | A0A0C5C9V4 | A0A3Q7H5I5 | A0A3Q7GKP3 | A0A3Q7IMV7 | A0A3Q7F8X4 |
| A0A3Q7J5Z9 | A0A3Q7JF75 | A0A3Q7EUQ6 | A0A3Q7GD09 | Q9AXQ5 | A0A3Q7EU87 | A0A3Q7GKR4 |
| A0A3Q7GDA5 | A0A3Q7IQB7 | A0A3Q7JGP1 | A0A3Q7IF15 | A0A3Q7I9J3 | A0A3Q7HU84 | P36181 |
| C6K2L0 | A0A3Q7H9K1 | A0A3Q7F246 | A0A3Q7J1A5 | A0A3Q7ERV5 | A0A3Q7GHS2 | Q38MV0 |
| A0A3Q7GY58 | Q7XBB3 | A0A3Q7J5P7 | B1Q3F1 | A0A3Q7GYN8 | A0A3Q7EYC4 | A0A3Q7JDF2 |
| A0A3Q7FZP1 | A7LI54 | A0A3Q7I4U3 | A0A3Q7FSY4 | A0A3Q7FLA3 | A0A3Q7JEN8 | A0A3Q7GYY4 |
| A0A3Q7H4R7 | A0A3Q7HUE5 | C0LIR3 | K4ATQ2 | Q10712 | A0A3Q7GL30 | A0A3Q7GAU9 |
| A0A3Q7IKN3 | A0A3Q7G5T0 | P43280 | A0A3Q7HEX8 | A0A3Q7HC95 | A0A3Q7ESA9 | A0A3Q7JQE3 |
| A0A3Q7GSL2 | A0A3Q7HD29 | Q8GZR6 | A0A3Q7G8L2 | A0A3Q7EXW0 | A0A3Q7FY03 | A0A3Q7I956 |
| A0A3Q7G720 | C6K2K9 | A0A3Q7F6N7 | P93841 | A0A3Q7HY56 | A0A3Q7INL4 | Q5NE21 |
| A0A3Q7ILV3 | A0A3Q7FA43 | A0A3Q7FF30 | A0A3Q7EJA6 | A0A3Q7F579 | P08706 | A0A3Q7FPY3 |
| A0A3Q7H3C5 | A0A3Q7I412 | A0A3Q7HRB1 | A0A3Q7FAE2 | A0A3Q7JP21 | A0A3Q7FR28 | A0A3Q7J2T1 |
| Q94K24 | A0A3Q7HZM0 | A0A3Q7H1W0 | A0A3Q7FKJ0 | A0A3Q7IXE6 | G8Z254 | A0A3Q7H001 |
| A0A3Q7HV28 | A0A3Q7IA38 | A0A3Q7ENN1 | A0A3Q7G863 | A0A3Q7I9R7 | A0A3Q7HAN1 | A0A3Q7HA43 |
| A0A3Q7IKB1 | P49297 | A0A3Q7IBT3 | A0A3Q7GEV3 | A0A3Q7H7F3 | A0A3Q7EQN7 | A0A3Q7HGQ5 |
| K4BAE6 | A0A3Q7GLM4 | A0A3Q7HLK8 | A0A3Q7HM51 | A0A3Q7I5A7 | A0A3Q7I7B3 | A0A3Q7EY39 |
| A0A3Q7EZJ3 | A0A3Q7IGR5 | Q8GZD8 | A0A3Q7F639 | A0A3Q7EHT5 | Q8GT30 | A0A3Q7E8X9 |
| A0A3Q7HSR7 | A0A3Q7HRQ8 | A0A3Q7FTI8 | A0A3Q7HGJ9 | Q8RU74 | A0A3Q7F395 | A0A3Q7HZQ4 |
| A0A3Q7HVI4 | A0A3Q7FE34 | Q9ZS45 | A0A3Q7EQ78 | A0A3Q7HVD5 | A0A3Q7HGG4 | A0A3Q7IQV2 |
| A0A3Q7ETU0 | A0A3Q7IKX3 | A0A3Q7ER22 | A0A3Q7ERM4 | P93541 | A0A3Q7J6X7 | A0A3Q7IQZ0 |
| O49877 | Q8H6B5 | A0A3Q7F897 | A0A3Q7FFV1 | Q9XG54 | A0A3Q7GB69 | A0A3Q7GQZ9 |
| C0KKU8 | A0A3Q7GYB4 | A0A3Q7JPE6 | A0A3Q7HTM2 | A0A3Q7H3K1 | A0A3Q7G427 | A0A3Q7FL73 |
| A0A3Q7F980 | A0A3Q7FJZ3 | K4AT92 | A0A3Q7IPK7 | A0A3Q7F7I4 | A0A3Q7HVG8 | Q941R1 |
| A0A3Q7H4V4 | A0A3Q7FPW5 | A0A3Q7IH89 | A0A3Q7FVL0 | A0A3Q7J3Z0 | A0A3Q7FU38 | A0A3Q7G4W7 |
| A0A3Q7JFF5 | A0A3Q7JE58 | A0A3Q7GDK7 | A0A3Q7JBP6 | A0A3Q7EUS0 | A0A3Q7H7H5 | A0A3Q7G744 |
| A0A3Q7IP06 | A0A3Q7H940 | A0A3Q7F3P8 | Q9THX6 | A0A3Q7HGQ2 | P31542 | A0A3Q7IT51 |
| A0A220QMI6 | A0A3Q7GX88 | Q9FEW9 | A0A3Q7F529 | A0A3Q7GWP0 | A0A3Q7FTM5 | A0A3Q7GXH5 |
| A0A3Q7HBV3 | A0A3Q7FW96 | Q672Q6 | A0A3Q7FVD3 | A0A3Q7H4P9 | A0A3Q7I008 | A0A3Q7H1P7 |
| A0A3Q7HFB9 | A0A3Q7HIS1 | P21568 | A0A3Q7IA36 | A0A3Q7HS42 | A0A3Q7FMF3 | Q5NE20 |
| A0A3Q7EHM0 | A0A3Q7F2F3 | Q3I5C4 | A0A3Q7IRF8 | A0A0C5CHB1 | A0A3Q7HVF4 | A0A3Q7F9P6 |
| A0A3Q7JSU0 | A0A3Q7JD34 | A0A3Q7GCR0 | A0A3Q7HBR5 | A0A3Q7F8X2 | A0A3Q7HRU2 | A0A3Q7FEB3 |
| K4CLT6 | B2MWN0 | A0A3Q7HV43 | A0A3Q7G667 | A0A3Q7FS97 | A0A3Q7J565 | A0A3Q7HZE0 |
| A0A3Q7I7H7 | E5LBC4 | A0A3Q7FTZ2 | A0A3Q7FZI5 | A0A3Q7FNI9 | A0A3Q7F3Y2 | A0A3Q7FYP5 |
| A0A3Q7IQK0 | A0A3Q7IAU4 | A0A3Q7FBR4 | A0A3Q7F0L3 | A0A3Q7EPW1 | A0A3Q7HC56 | A0A3Q7EHY8 |
| A0A3Q7FNL7 | A0A3Q7EI69 | A0A3Q7F6F6 | A0A3Q7FEL1 | A0A3Q7F3S5 | A0A3Q7IRF9 | A0A3Q7HVI2 |
| A0A3Q7HWQ1 | A0A3Q7I0E4 | A0A3Q7INM0 | A0A3Q7FSH7 | Q40129 | A0A3Q7JEL1 | A0A3Q7IPV0 |
| A0A3Q7EN75 | A0A3Q7IZ37 | A0A3Q7FW72 | A0A3Q7ISE3 | A0A3Q7GJA8 | Q9FYW9 | A0A3Q7GWC2 |
| A0A3Q7FKF5 | A0A3Q7GYE7 | A0A3Q7GQR0 | A0A3Q7H5X9 | A0A3Q7GKL8 | A0A3Q7GE03 | G8Z261 |
| A0A3Q7G5P1 | A0A3Q7EHY3 | A0A3Q7H0H2 | A0A3Q7FQ28 | A0A3Q7I9N3 | A0A3Q7FWS2 | Q94FW7 |
| B1Q3F8 | A0A3Q7FW21 | A0RZD0 | K4B3H9 | A0A3Q7IKR1 | A0A3Q7IL22 | Q9FUZ0 |
| A0A3Q7IM38 | A0A3Q7HGY9 | A0A3Q7HU15 | A0A3Q7GG75 | A0A3Q7E6Y7 | A0A3Q7EM11 | A0A0C5CHA6 |
| Q9LEG5 | A0A3Q7IJ63 | A0A3Q7HXK5 | Q93X44 | A0A3Q7GR78 | Q3SC85 | A0A3Q7H097 |
| A0A3Q7I7T0 | Q8RXB7 | P49118 | A0A3Q7GYF2 | A0A3Q7J5K6 | A0A3Q7GP46 | A0A3Q7IHG0 |
| A0A3Q7F0F8 | A0A3Q7EYM6 | A0A3Q7FU99 | A0A3Q7I5G1 | A0A3P3ZKL1 | A0A3Q7GA27 | A0A3Q7JX44 |
| A0A3Q7JCM5 | A0A3Q7IUK7 | A0A3Q7FZA2 | A0A3Q7FZI2 | A0A3Q7ESN3 | Q05538 | A0A3Q7IIB9 |
| C5IU71 | A0A3Q7ICF8 | A0A3Q7IGH0 | A0A3Q7IQ38 | A0A3Q7FB69 | A0A3Q7F0P2 | A0A3Q7FRC7 |
| A0A3Q7EPC2 | A0A3Q7ES85 | A0A3Q7ESL5 | A0A3Q7J583 | A0A3Q7IIH1 | A0A3Q7GWB4 | A0A3Q7GDG7 |
| A0A3Q7F479 | A0A3Q7EF29 | A0A3Q7HXP3 | A0A3Q7GPY6 | A0A3Q7J408 | A0A3Q7H4F2 | A0A3Q7H1U4 |
| A0A3Q7JDP0 | A0A3Q7ECG0 | A0A3Q7FKE1 | A0A3Q7J2P9 | A0A3Q7ESI6 | A0A3Q7FWZ9 | A0A3Q7EHH5 |
| A0A3Q7GEU9 | A0A3Q7HHA0 | A0A3Q7H441 | A0A3Q7IZR8 | A0A3Q7GEI3 | A0A3Q7IM99 | A0A3Q7JEE7 |
| A0A3Q7H1Q5 | A0A3Q7IMU3 | K4D2A4 | P37218 | Q672Q2 | A0A3Q7FKZ2 | A0A3Q7ENV3 |
| A0A3Q7FCJ1 | A0A3Q7HZ41 | K4B381 | K4C627 | A0A3Q7FXR7 | A0A3Q7I6Y9 | A0A3Q7GED7 |
| A0A3Q7IRR8 | A0A3Q7F289 | A0A3Q7GEZ4 | A0A3Q7EZW3 | A0A3Q7HFB7 | A0A3Q7IUI9 | A0A3Q7H1L5 |
| C6KI36 | A0A3Q7ITL4 | A0A3Q7GSF7 | A0A3Q7GPU8 | A0A3Q7FS41 | A0A3Q7F9B8 | A0A3Q7FYL1 |
| A0A3Q7H0W4 | A0A3Q7HV74 | A0A3Q7HNA9 | A0A3Q7HIS4 | A0A3Q7ER18 | P26300 | A0A3Q7I185 |
| A0A3Q7GLT1 | B5M9E4 | Q202I0 | A0A3Q7F7C5 | P93214 | P25306 | A0A3Q7EJ55 |
| A0A3Q7JDN9 | A0A3Q7H0E4 | A0A3Q7IAC1 | A0A0C5CEC3 | A0A3Q7EA02 | A0A3Q7FSE5 | A0A3Q7HUN1 |
| A0A3Q7G7Y1 | A0A3Q7INB5 | A0A3Q7ERM5 | A0A3Q7I6B9 | A0A3Q7EU50 | A0A3Q7EKI7 | A0A3Q7ENB7 |
| A0A3Q7EI59 | A0A3Q7J9P7 | A0A3Q7F8I3 | A0A3Q7F385 | A0A3Q7G5J6 | A0A3Q7GXR4 | A0A3Q7JFP9 |
| Q40131 | A0A3Q7ISK0 | A6N6K8 | A0A3Q7GH03 | A0A3Q7G4E2 | Q5NE17 | A0A3Q7EQJ0 |
| A0A3Q7ILY0 | A0A3Q7J519 | A0A3Q7JAV3 | A0A3Q7HGG5 | A0A3Q7FZG5 | A0A3Q7EQ38 | A0A3Q7IIH8 |
| A0A3Q7HQL1 | Q84XW6 | A0A3Q7JBV0 | A0A3Q7IXH4 | A0A3Q7G3B0 | A0A3Q7GAX3 | A0A3Q7ERH5 |
| A0A3Q7GAX9 | A0A3Q7HMP2 | P17786 | A0A3Q7IV51 | A0A3Q7IK00 | A0A3Q7HT73 | A0A3Q7HTH3 |
| A0A3Q7HRC2 | A0A3Q7GQU3 | A0A3Q7IS88 | G8D593 | A0A3Q7JXC3 | A0A3Q7E8H4 | A0A3Q7HUQ3 |
| H6WYS2 | A0A3Q7GQM4 | A0A3Q7GND4 | A0A3Q7JIX8 | A0A3Q7GBP8 | A0A3Q7HR44 | A0A3Q7GD18 |
| A0A3Q7E8I1 | A0A3Q7J418 | A0A3Q7G2Q9 | A0A3Q7HRC7 | A0A3Q7INV1 | A0A3Q7FWY8 | A0A3Q7IS73 |
| A0A3Q7HMJ3 | G3K2M4 | A0A3Q7IN43 | A0A3Q7G242 | A0A3Q7FB85 | A0A3Q7FS73 | A0A3Q7J0Q1 |
| A0A3Q7GS73 | A0A3Q7F9C8 | A0A3Q7GS13 | A0A3Q7F5D0 | B1Q3F7 | A0A3Q7H7Q7 | A0A3Q7F3X8 |
| A0A3Q7IBI0 | A0A3Q7G0X6 | A0A3Q7HLZ4 | A0A3Q7HJU0 | A0A3Q7EJL3 | Q9AXQ3 | A0A0C5CEG4 |
| A4ZYQ6 | C0LIR4 | A0A3Q7I8V2 | A0A3Q7EK01 | A0A3Q7IFL7 | K4BXV0 | A0A3Q7EUR6 |
| A0A3Q7GZW2 | A0A3Q7I3V5 | A0A3Q7EJS4 | A0A3Q7FWB5 | A0A3Q7IDX8 | A0A3Q7IB30 | P07179 |
| A0A3Q7ELA6 | A0A3Q7J3F5 | A0A3Q7EDX6 | A0A3Q7IP97 | A0A3Q7E9H0 | D0VNY3 | A0A3Q7JST6 |
| A0A3Q7J566 | A0A3Q7JYD7 | A0A3Q7HX95 | A0A3Q7JHP4 | A0A3Q7I0A1 | A0A3Q7GAE5 | A0A3Q7EXK0 |
| A0A3Q7ITD1 | A0A3Q7HZV3 | A0A3Q7E9C5 | A0A3Q7HK40 | A0A3Q7EN07 | A0A3Q7H0U0 | A0A3Q7ILF6 |

**Note: Arrange according to the corresponding protein entry number of Uniprot**

Supplemental Table 2 Primer sequences for cloning and related vector construction.

| **Assay** | **Name** | **Forward primer (5'-3')** | **Reverse primer (5'-3')** |
| --- | --- | --- | --- |
| **Gene clone** | MEK1 | ATGAAGAAAGGATCTTTTGCACCTAATCTTAAAC | TAGCTCAGTAAGTGTTGCCAATGG |
| **Super 1300-GFP** | MEK1-GFP | GAGCTCGGTACCCGGGGATCCATGAAGAAAGGATCTTTTGCACCT | CATGTCGACTCTAGAGGATCCTAGCTCAGTAAGTGTTGCCAATGG |
| **pET30a** | pET30a-MEK1 | GGAATTCATGAAGAAAGGATCTTTTGCACCTAATCTTAAAC | CCCAAGCTTTTATAGCTCAGTAAGTGTTGCCAATGG |
| **PAC004-HA** | MEK1-HA | TTACAATTACCATGGGGCGCGCCATGAAGAAAGGATCTTTTGCACCT | AACATCGTATGGGTAGGTACCTAGCTCAGTAAGTGTTGCCAATGG |
| **Crispr cas9** | MEK1Primer 1 | GA TTGAAGA TGGACAAGGCT | CTGGTCTCTTAAATGTTCCTGATTCAGTCTGCACCAGCCGGGAA |
|  | MEK1Primer 2 | GCTGGTCTCTTTTAGTTTTAGAGCTAGAAATAGCAAGTTA | GCTGGTCTCTAAACGCCGCACTATACCACCATTTTGCACCAGCCGGGAATCG |
| **Interaction** | Luc | GGGGTACCATGAAGAAAGGATCTTTTGCACCTAATCTTAAAC | GCGTCGACTAGCTCAGTAAGTGTTGCCAATGG |
|  | Bifc | GGGGTACCATGAAGAAAGGATCTTTTGCACCTAATCTTAAAC | GCGTCGACTAGCTCAGTAAGTGTTGCCAATGG |
|  | Y2H | GGAATTCATGAAGAAAGGATCTTTTGCACCTAATCTTAAAC | GCGTCGACTTATAGCTCAGTAAGTGTTGCCAATGG |
| **Mutant seedlings** | Cri-MEK1 | ATGAAGAAAGGATCTTTTGCACCTA | AATATACCTTGAGAGCGAAAAATTGC |

Supplemental Table 3 Primers sequences used for qRT-PCR assays.

| Gene name | Forward primer (5'-3') | Reverse primer (5'-3') |
| --- | --- | --- |
| *Slactin* | AATGAACTTCGTGTGGCTCCAGAG | ATGGCAGGGGTGTTGAAGGTTTC |
| *SlGSNOR* | ATGGCTACACAAGGTCAAG | GTCCTGGATATGTTTGTATGA |
| *SlMEK1* | ACAGCCATCAGACAACCAGTTATGC | GCCGCACTATACCACCATTTCCC |
